# Group A *Streptococcus* interacts with glycosaminoglycans via M proteins to modulate bacterial adherence *in vitro*

**DOI:** 10.1111/febs.70167

**Published:** 2025-07-03

**Authors:** Tahnee B.‐D. McEwan, David M. P. De Oliveira, Emily K. Stares, Lauren E. Hartley‐Tassell, Christopher J. Day, Mark J. Walker, Michael P. Jennings, Ronald Sluyter, Martina L. Sanderson‐Smith

**Affiliations:** ^1^ Molecular Horizons and School of Science University of Wollongong Wollongong Australia; ^2^ Institute for Molecular Biosciences, The Centre for Superbug Solutions The University of Queensland Brisbane Australia; ^3^ Institute for Biomedicine and Glycomics Griffith University Gold Coast Australia; ^4^ Present address: The Australian Arthritis and Autoimmune Biobank Collaborative (A3BC) and the Sutton Arthritis Research Laboratory, Sydney Musculoskeletal Health, The Kolling Institute of Medical Research, The University of Sydney and the Northern Sydney Local Health District Sydney Australia; ^5^ Present address: Institute for Molecular Biosciences, The Centre for Superbug Solutions The University of Queensland Brisbane Australia; ^6^ Present address: Teva Pharmaceuticals Sydney Australia

**Keywords:** epithelial cells, flow cytometry, glycan microarray, polysaccharides, *Streptococcus pyogenes*

## Abstract

Glycosaminoglycans (GAGs) are enriched in the cutaneous extracellular matrix and have important roles in bacterial colonisation. Group A *Streptococcus* (GAS) can be categorised by *emm* patterning and M‐family protein expression. M proteins of GAS are major adhesins with lectin‐binding properties. This study aimed to provide a comprehensive specificity and affinity profile of phylogenetically diverse M proteins to a range of sulfated host GAGs and to investigate the physiological relevance of these interactions. Chondroitin sulfate preferentially associated with M proteins of A–C pattern strains, with binding localised to the central variable region of M1 protein. Dermatan sulfate was shown to associate with M proteins of all pattern type strains, with recognition involving multiple sites on M proteins. Heparin and heparan sulfate exclusively interacted with M proteins of A–C and D pattern strains. Multiple sites of M proteins were involved in heparin recognition, as indicated by surface plasmon resonance and site‐directed mutagenesis of the heparin‐binding XBXBX motif in the hypervariable‐central region of M53 protein. In contrast, binding of heparan sulfate was localised to the non‐repeat region between the B2 repeat and C1 repeat of M53 proteins. 5448 (M1‐expressing GAS, A–C pattern) was shown to bind chondroitin sulfate, dermatan sulfate and heparin in an M protein‐dependent manner. Furthermore, recruitment of chondroitin sulfate or dermatan sulfate by M1 proteins, but not heparin, was shown to increase GAS adherence to human HaCaT keratinocytes. This study increases our understanding of the molecular mechanisms underlying GAS adhesion, with key implications for bacterial colonisation and persistence of infection.

## Graphical abstract

Interactions between bacteria and the extracellular matrix are a prerequisite for colonisation and infection. Phylogenetically diverse M proteins of group A *Streptococcus* (GAS) can selectively bind sulfated glycosaminoglycans with high affinity. M protein‐mediated recruitment of chondroitin sulfate and dermatan sulfate was shown to increase GAS adherence to HaCaT keratinocytes. These findings could lead to new strategies for preventing or treating GAS infections by blocking these attachment processes.

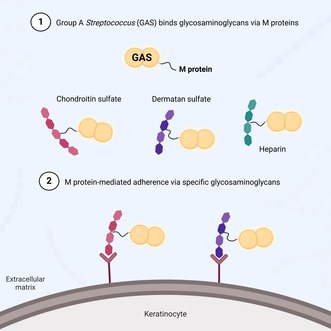

**Abbreviations**
ECM: extracellular matrix
GAG: glycosaminoglycan
GAS: group A *Streptococcus*
IdoA: iduronic acid
*K*_D_
: equilibrium dissociation constant
MOI: multiplicity of infection
NTA: nitrilotriacetic acid
RC: reverse complement
SEM: standard error of the mean
SPR: surface plasmon resonance

## Introduction

Glycosaminoglycans (GAGs) are negatively charged mucopolysaccharides characterised by linear chains of repeating disaccharide units and are enriched in the extracellular matrix (ECM) of various host tissue and bodily fluids, and the pericellular space where they are found on the cell surface as components of proteoglycans [1]. Each disaccharide unit consists of a uronic sugar or galactose covalently linked to an amino sugar by a glycosidic bond. The structural composition of GAGs has given rise to functionally distinct subclasses consisting of heparin, heparan sulfate, dermatan sulfate, chondroitin sulfate, hyaluronic acid and keratan sulfate [2]. Heterogeneity within these classes arises from chain length, sulfation pattern and/or epimerisation which contributes to the challenging nature of studying the specificity of GAG interactions.

Inflammatory mediators such as cytokines, and reactive oxygen species from immune cells during infection can alter the surface expression of GAGs by disrupting GAG metabolism [3, 4] which can be exacerbated by the release of bacterial‐derived enzymes or GAG‐specific lyases [5]. Dermatan sulfate can be liberated from proteoglycans present in human primary skin fibroblasts by the action of secretory cysteine proteases from group A *Streptococcus* (GAS; *Streptococcus pyogenes*), a deadly causative agent of skin, soft tissue and respiratory tract infections [6], which can subsequently bind and neutralise the bactericidal activity of α‐defensins released by neutrophils [7]. This represents a mechanism of GAS immune evasion governed by the exploitation of host GAGs.

There is increasing evidence to suggest GAGs are exploited in the early stages of GAS infection for adhesion preceding host injury or stimulation of the immune response. Early investigations found that exogenous heparin and heparan sulfate could selectively inhibit the adhesion of GAS protein extract to monkey cardiac muscle and kidney tissue [8], suggesting the existence of GAG‐specific lectins. Later studies also revealed that exogenous dermatan sulfate and heparan sulfate could decrease GAS adherence to Detroit 562 pharyngeal cells and human primary foreskin fibroblasts [9]. Targeted enzymatic cleavage of endogenous heparan sulfate, chondroitin sulfate and dermatan sulfate from various host cell types, or inclusion of GAG synthesis or sulfation inhibitors, has also been shown to decrease GAS adherence [9, 10], providing further evidence that host GAGs can function as ligands to mediate GAS adhesion to host cells.

Decorating the surface of GAS strains are M proteins, encoded by the *emm* gene. The diverse arrangement of *emm* family genes serves as systematic tool for classifying GAS strains into five *emm* patterns (A–E). M proteins can function as a promiscuous adhesin and lectin, serving as binding receptors to host glycans and GAGs such as human blood group antigens [ABO(H)] [11, 12], dermatan sulfate [9] and hyaluronic acid [13] on a protein and whole cell level. Recent work has provided a comprehensive binding and functional analysis of M protein interactions with hyaluronic acid, the only non‐sulfated GAG, and found that hyaluronic acid increased GAS adherence to HaCaT keratinocytes independently of M proteins [13]. While there is some evidence to support a role for M proteins functioning as sulfated GAG receptors to enhance GAS‐mediated host cell adhesion [9], the diversity of both M proteins and GAGs involved has yet to be fully explored.

Thus, the aims of this study were to establish a binding selectivity profile of M protein interactions with sulfated GAGs and to investigate the contribution of these interactions in GAS adherence to HaCaT keratinocytes.

## Results

### GAG binding is a conserved function across phylogenetically diverse M proteins

**Fig. 1:**
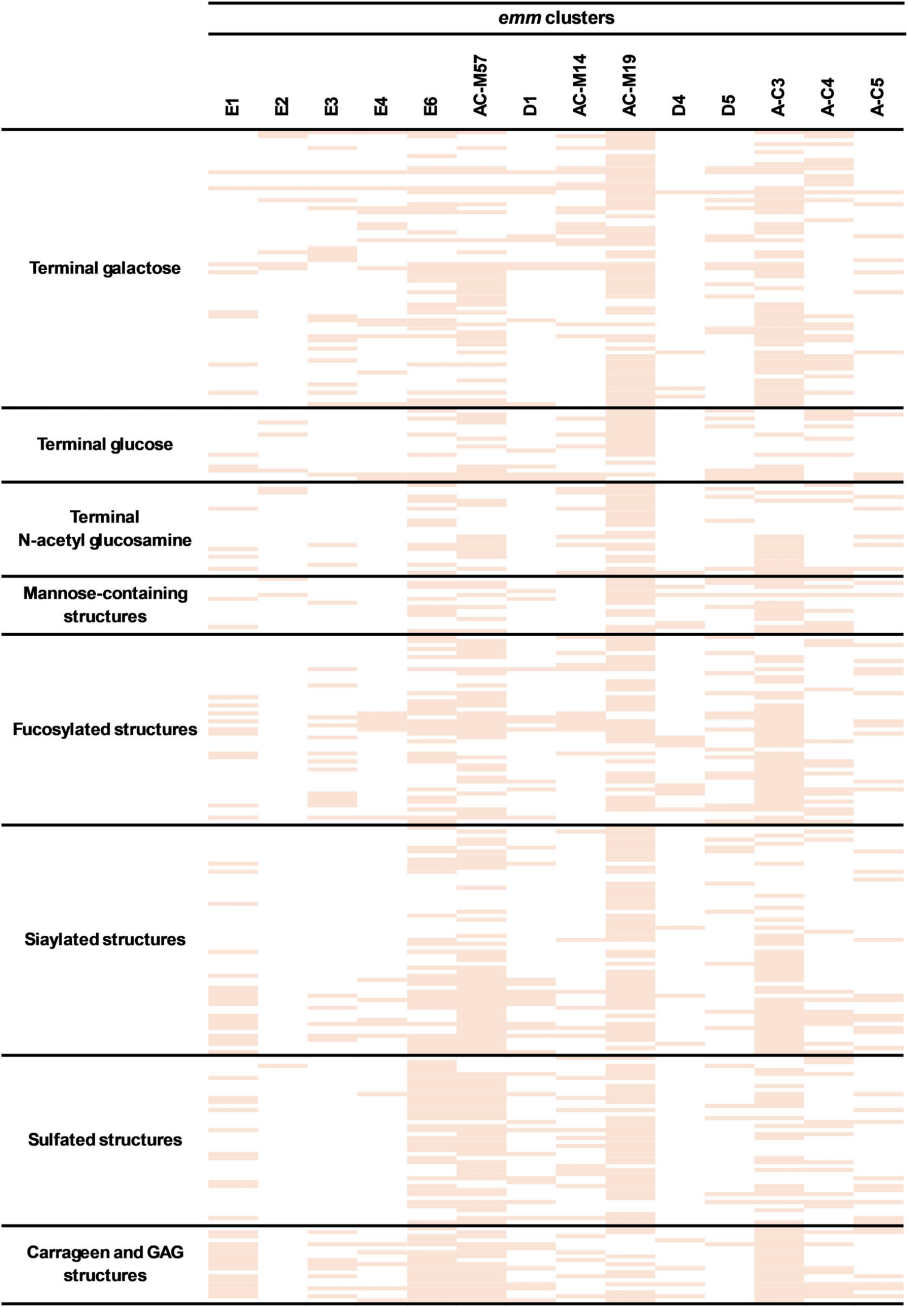
GAG binding is a conserved function across phylogenetically clustered M proteins. Binding profile of diverse glycan structures with phylogenetically clustered M protein using a glycan microarray. Orange blocks, indicative of glycan binding, are presented only if all M protein representatives within a cluster group (M1, M2, M3, M9, M11, M12, M14, M19, M53, M54, M57, M58, M60, M65, M70, M90, M97, M98, M102, M106) were able to bind the respective glycan structure from three independent experiments.

Advancements in glycomics have led to the development of glycan microarray technology which facilitates a rapid screening of glycan interactions with potential lectins [14, 15]. Hence, a comprehensive glycan binding profile was established using different M‐types from 11 major *emm*‐clusters as well as non‐clustered M protein (Clade Y, A‐C; M57, M14, M19), against a panel of 287 distinct glycan structures including sulfated GAGs (Tables S1 and S2) [13]. Recombinant M protein representatives from each *emm‐*cluster were shown to have distinct glycan binding profiles, with several *emm*‐cluster groups demonstrating broad specificity for multiple terminal galactose, glucose, fucose, mannose, sialic acid and GAG‐containing structures (Fig. 1).

Glycan array analysis revealed that binding of heparin (Glycan index 298; [16]) and unsaturated heparin disaccharides (Glycan indexes 279–280; 282–283) was a shared function among most M protein cluster groups. Only M proteins representative of clusters E2, E4 and D5 did not exhibit conserved heparin and digested heparin fragment binding function. In contrast, binding of heparan sulfate (Glycan index 314) and unsaturated heparan sulfate disaccharides (Glycan indexes 279–280; 282–283) was only observed for clusters E1, E4, E6, D1, A‐C3 as well as non‐clustered AC‐57 and AC‐19 M protein. Unsaturated disaccharides that can be produced from either heparin or heparan sulfate (Glycan indexes 281 and 284) resulted in binding to most M protein cluster groups excluding E2, D1, D4, D5 and A‐C4. Together, the evidence suggests that M proteins representative of E2 or D5 cluster groups do not exhibit heparin or heparan sulfate binding function. When compared to 6‐*O*‐monosulfated chondroitin sulfate (Glycan index 301), 4,6‐*O*‐disulfated chondroitin sulfate (Glycan index 299) lost a capacity to bind cluster D4 M protein as well as non‐clustered AC‐M14 and AC‐M19 proteins, instead facilitating binding to cluster E4 and D1 M protein. Both chondroitin sulfate subtypes bound clusters E1, E3, E6, A‐C3, A‐C4, A‐C5, as well as non‐clustered AC‐M57 protein. It should be noted that glycan indexes 299 and 301 may reflect noncanonical chondroitin sulfate/dermatan sulfate hybrids, as minor iduronic acid (IdoA) substitutions can arise from partial enzymatic epimerisation in biological preparations [17], a modification that could have contributed to the observed binding interaction profiles. Binding of dermatan sulfate (Glycan index 300) was restricted to M proteins representative of clusters E1, E6, A‐C3, A‐C4 as well as non‐clustered AC‐M57 and AC‐M19 proteins. Collectively, this dataset revealed that different GAGs can bind to phylogenetically diverse M proteins and suggests that GAG binding is a conserved function across *emm* types that may be dependent on polysaccharide composition and sulfation pattern.

### M proteins bind to GAGs with high affinity

**Fig. 2:**
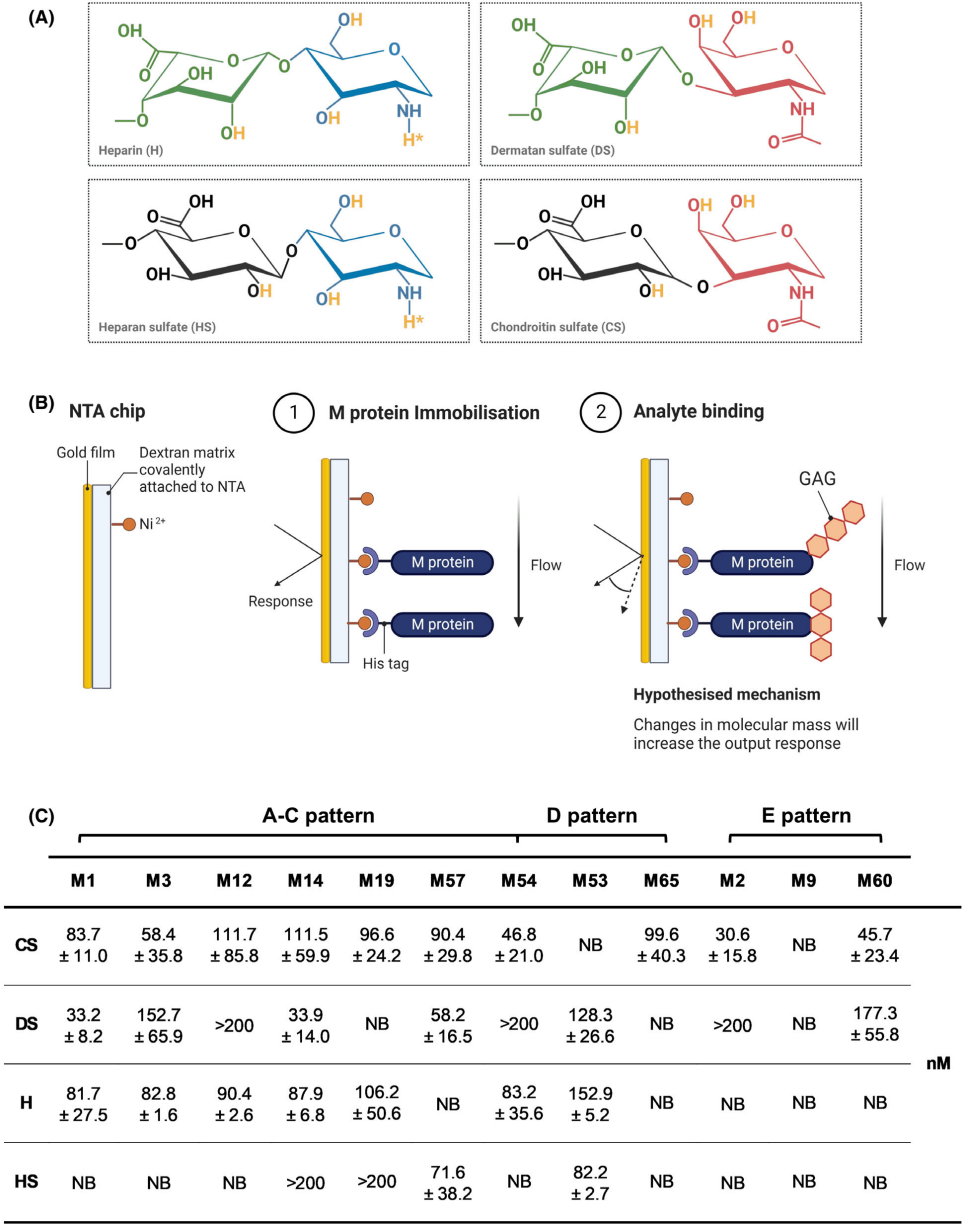
M proteins bind to GAG subclasses with high affinity. (A) Representative disaccharide structures of heparin (H), dermatan sulfate (DS), heparan sulfate (HS) and chondroitin sulfate (CS) are shown in variably substituted forms, rather than as native unmodified monomers. Heterogeneity arises from chain length, sulfation pattern and/or epimerisation. Each disaccharide unit consists of a uronic sugar (D‐glucuronic acid, black; L‐iduronic acid, green) that is linked to an amino sugar (glucosamine, blue; N‐acetylgalactosamine, red). Sites that may be substituted with sulfate moieties (−SO_3_^−^) are shown in yellow. Sites that may be substituted with an acetyl group (−COCH_3_) are denoted with an asterisk (*). Created with BioRender. (B) Schematic of the surface plasmon resonance (SPR) approach utilised for assessing M protein–glycosaminoglycan (GAG) binding interactions. NTA, nitrilotriacetic acid. Created with BioRender. (C) Equilibrium dissociation constants (*K*_D_: nm) for M protein–GAG interactions determined using SPR. NB, non‐binding. Data shown are mean ± SEM from three independent experiments.

To partially validate the glycan microarray dataset and establish a comprehensive specificity and affinity profile of M protein interactions with a range of sulfated GAGs (Fig. 2A), surface plasmon resonance (SPR) was utilised to monitor interactions and establish binding affinities in real time. Phylogenetically diverse M proteins were non‐covalently immobilised onto activated nitrilotriacetic acid (NTA) sensors in a physiologically relevant orientation (Fig. 2B; [13]) and GAG binding was assessed using single‐cycle kinetics with equilibrium dissociation constants (*K*_D_) determined for each interaction. Chondroitin sulfate was demonstrated to preferentially associate with M proteins of A‐C pattern strains, binding to all M proteins except for M53 (pattern D) and M9 (pattern E) protein with high affinity (30.6–111.7 nm; Fig. 2C; Fig. S1). Dermatan sulfate was shown to associate with M proteins of all pattern type strains including M1 protein as previously reported [9]; however, binding to M19, M65 and M9 protein was not observed in the tested concentration range. Of the M proteins that bound dermatan sulfate, equilibrium dissociation constants ranged between 33.2–177.3 nm (Fig. 2C; Fig. S2). Both heparin and heparan sulfate exclusively interacted with M proteins of A‐C and D pattern strains; albeit, more M proteins could interact with heparin than heparan sulfate. For M proteins that bound heparin, equilibrium dissociation constants ranged between 81.7–152.9 nm (Fig. 2C; Fig. S3). Heparan sulfate binding was restricted to M14, M19, M57 and M53 protein, with the latter two M proteins revealing equilibrium dissociation constants of 71.6 and 82.2 nm respectively (Fig. 2C; Fig. S4). M protein–GAG interactions where equilibrium dissociation constants could not be determined (*K*_D_ > 200 nm) were not further investigated in this study. Collectively, these data indicate that phylogenetically diverse M proteins can bind various host GAGs with high affinity, with evidence of conserved or M protein‐specific functions.

**Fig. 3:**
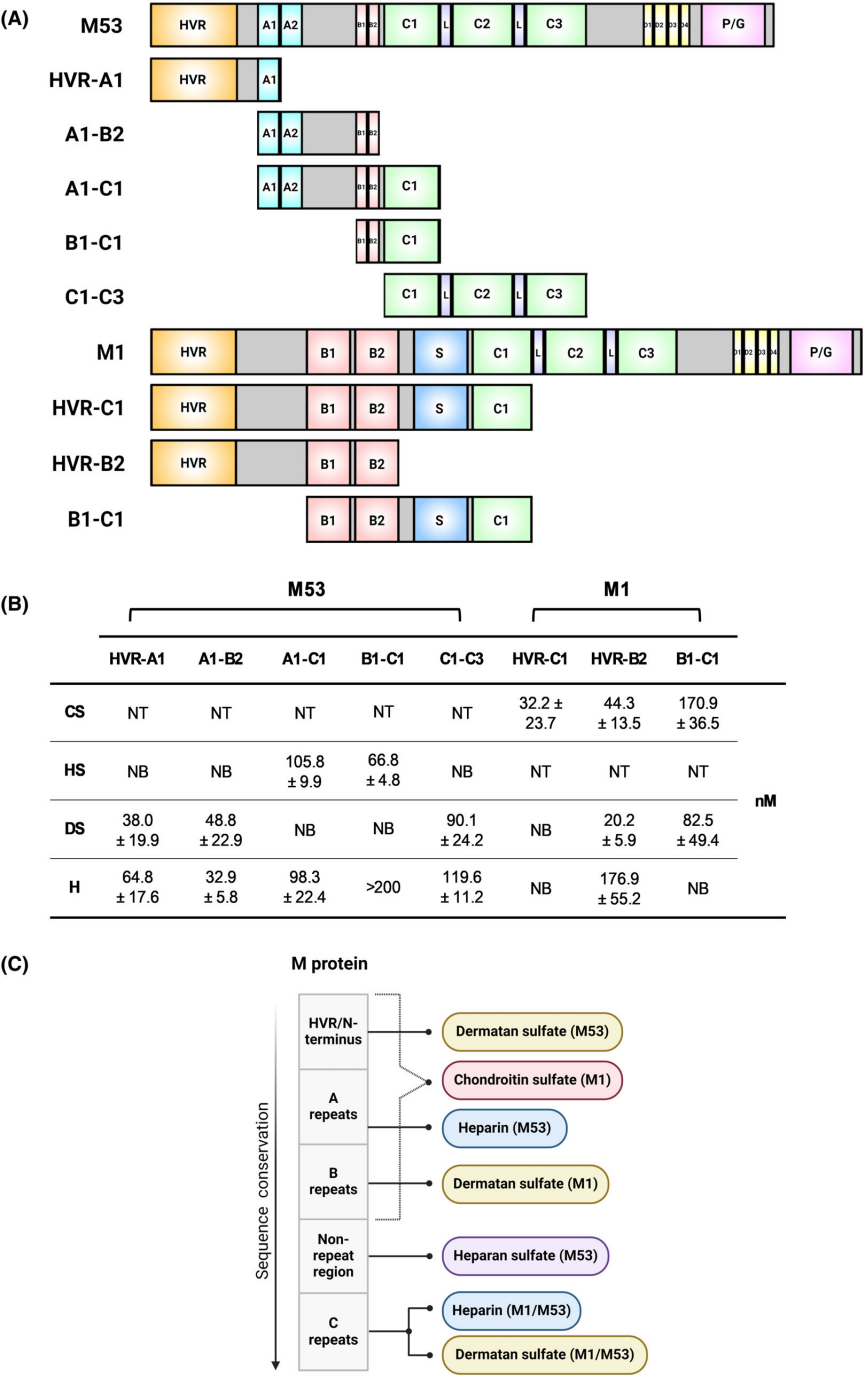
Discrete repeat domains between phylogenetically diverse M proteins underlie GAG recognition. (A) Fragments of M53 and M1 proteins were designed with overlapping repeat domains. GenBank accession numbers used for structural domain mapping are M53 (P49054.1) and M1 [WP_011285743.1 or CP008776.1 (5448) and JX028599.1], visualised using Illustrator for Biological Sequences and BioRender, where the size of each M protein and the respective domains are to scale. M53 and M1 proteins are structurally organised by four repeat domain regions (A–D) arranged in tandem where the number, size and amino acid composition of these regions give rise to heterogeneity. The hypervariable region (HVR) lies at the N terminus, followed by the central variable region, with the proline/glycine (P/G)‐rich region at the highly conserved C terminus. Linker (L) regions support the C repeat regions and the M1 protein possesses a unique S‐region. (B) Equilibrium dissociation constants (*K*_D_: nm) for M protein fragment interactions with chondroitin sulfate (CS), heparan sulfate (HS), dermatan sulfate (DS) and heparin (H) using surface plasmon resonance (SPR). (C) Summary of glycosaminoglycan (GAG)‐binding domains on M proteins determined by SPR and visualised using BioRender. NB, non‐binding; NT, not tested. (B) Data shown are mean ± SEM from three independent experiments.

To identify specific binding domains in M proteins responsible for GAG recognition, truncation mutagenesis of phylogenetically diverse M53 and M1 proteins was conducted, producing fragments with overlapping repeat domains as previously characterised ([11, 13]), and binding of truncated proteins to GAGs was assessed via SPR analysis (Fig. 3A). As chondroitin sulfate was shown to bind full‐length M1 but not M53 protein, only the binding of chondroitin sulfate to M1 protein fragments was assessed. Chondroitin sulfate was shown to bind HVR‐C1 and HVR‐B2 fragments with higher affinity than the B1‐C1 fragment (32.2 nm and 44.3 nm < 170.9 nm) (Fig. 3B; Fig. S5), suggesting a chondroitin sulfate‐binding motif may be localised in the central variable region of M1 proteins. Since heparan sulfate was shown to bind full‐length M53 protein but not M1 protein, only the binding of heparan sulfate to M53 protein fragments was examined. Binding of heparan sulfate to the A1‐C1 and B1‐C1 fragments was observed with equilibrium dissociation constants of 105.8 and 66.8 nm, respectively (Fig. 3B; Fig. S6). In contrast, HVR‐A1, A1‐B2 and C1‐C3 fragments did not bind heparan sulfate (Fig. 3B). Together, these data indicate that the binding region for heparan sulfate may span the non‐repeat region situated between the B2 repeat and C1 repeat of the M53 protein.

Next, binding of dermatan sulfate to M53 and M1 protein fragments was assessed. Dermatan sulfate was shown to bind M53 (HVR‐A1, A1‐B2 and C1‐C3) protein fragments with high affinity (38.0–90.1 nm), while no binding was observed for M53 (A1‐C1) or M53 (B1‐C1) fragments (Fig. 3B; Fig. S7). Therefore, multiple dermatan sulfate‐binding motifs may exist at both the N terminus (hypervariable to A repeat region) and C terminus (C repeat region) of M53 protein, corresponding to previous reports using M5 protein [9]. For M1 protein fragments, dermatan sulfate was shown to bind to M1 (HVR‐B2) and M1 (B1‐C1) fragments with high affinity (20.2 and 82.5 nm respectively); however, no binding was observed with M1 (HVR‐C1) fragment (Fig. 3B; Fig. S7), but this may indicate differences in conformational dynamics between fragments. Based on the overlapping regions that bound dermatan sulfate, combined with the binding data of full‐length M1 protein described above, these data suggest the B repeat or C repeat region may be responsible for the binding of dermatan sulfate to M1 protein.

Similar to dermatan sulfate, heparin binding was shared by both M53 and M1 proteins. Hence, heparin binding to M53 and M1 protein fragments was explored. All M53 protein fragments were shown to bind heparin with varying affinities (32.9–119.6 nm) (Fig. 3B; Fig. S8). Fragments containing A repeats demonstrated high affinity interactions indicating this region may be primarily responsible for heparin binding to M53 protein. Given the C repeat region alone could also accommodate lower affinity interactions, the data suggest additional involvement of this region in heparin binding to M53 proteins. For M1 protein fragments, heparin was shown to only bind to the M1 (HVR‐B2) fragment with an equilibrium dissociation constant of 176.9 nm (Fig. 3B; Fig. S8), indicating the hypervariable region may be responsible for the binding of heparin although unlikely given the HVR‐C1 fragment did not bind heparin. Alternatively, this binding data may indicate the exposed B2 repeat may accommodate heparin binding specific to the M1 (HVR‐B2) fragment while the C repeat region may accommodate heparin binding in full‐length M1 protein, similar to M53 protein. A summary of the predominant GAG binding domains on M proteins determined by SPR in this study is provided (Fig. 3C).

### Partial disruption of the XBXBX heparin‐binding motif suggests a cooperative binding mechanism between M proteins and heparin

**Fig. 4:**
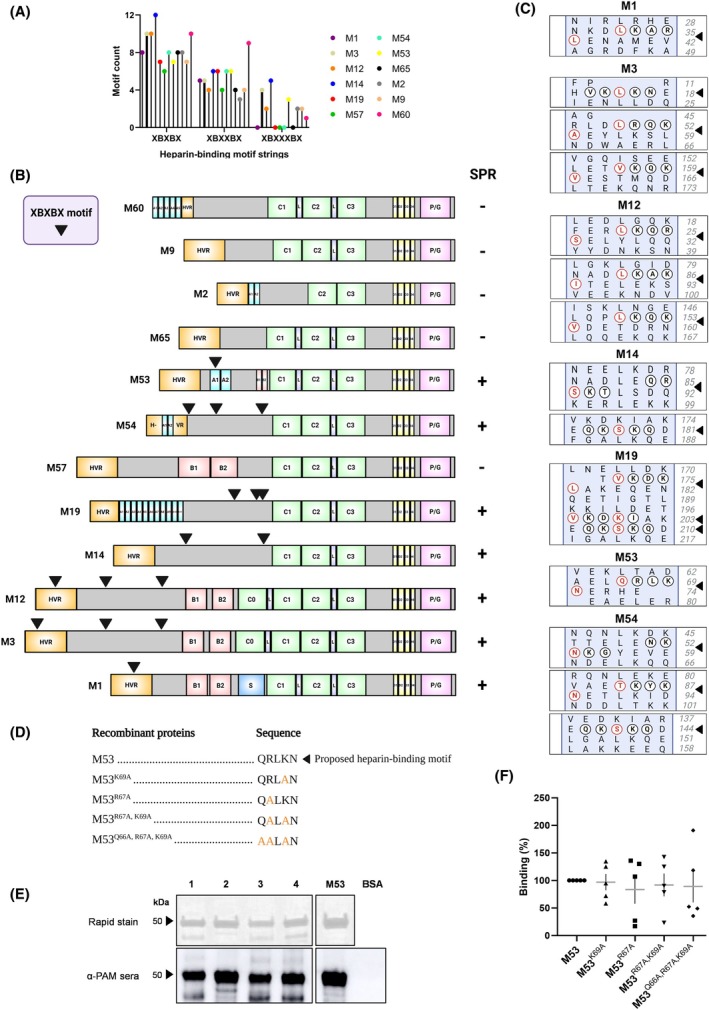
Targeted mutation of the XBXBX motif in M53 proteins does not significantly alter heparin binding. (A) Frequency of the top three heparin‐binding motif strings in M proteins where B represents any basic residue and X represents any other amino acid residue. (B) Frequency and distribution of refined XBXBX motifs in M proteins correlates with surface plasmon resonance (SPR) binding analysis. Arrows indicate specific positions of motifs on M proteins. Plus (+) denotes heparin binder. Minus (−) denotes non‐heparin binder. Refer to Fig. 3 for the structural domains of M proteins. (C) Heptad registers for each M protein were predicted using a Marcoil algorithm. Amino acid residues of the predicted heparin‐binding motif sequence present in M proteins are circled as indicated by black arrows. Red circled residues are buried in each coiled‐coil. Residue positions are indicated on the right. (D) Design of site‐directed M53 protein mutants. Residues within the proposed heparin‐binding site in M53 protein were substituted with alanine using site‐directed mutagenesis. Mutated residues are indicated in orange. (E) Concentrated purified site‐directed mutant M53 proteins (2 μg) were loaded onto a 10% polyacrylamide gel and electrophoresed under reducing conditions. Proteins were visualised using Rapid stain (top panel). Immunoblotting of proteins (200 ng) was performed using α‐PAM sera (1 : 30 000) to confirm identity (bottom panel). (1) M53^Q66A,R67A,K69A^; (2) M53^R67A,K69A^; (3) M53^R67A^; (4) M53^K69A^. Wild‐type M53 protein and bovine serum albumin were included as a positive and negative controls for sera specificity, respectively. (F) Binding of fluorescein‐labelled heparin to immobilised site‐directed mutant M53 proteins was measured and normalised to wild‐type M53 protein. Specific binding was determined by pre‐incubating with unlabelled GAG in excess. (E, F) Data shown are (E) representative from three independent experiments, or (F) mean ± SEM from five independent experiments and analysed using a one‐way analysis of variance (*P* > 0.05).

It is known that conserved linear binding motifs may underlie GAG recognition in proteins. A screening of 200 heparin‐binding proteins revealed that the top three heparin‐binding consensus sequences were XBXBX, XBXXBX, and XBXXXBX, where B represents any basic residue and X represents any other amino acid residue [18]. Therefore, the occurrence of these motif strings was examined in M protein sequences, and it was found that all M proteins contained multiple counts of XBXBX and XBXXBX motifs compared to the XBXXXBX motif (Fig. 4A). Focusing on XBXBX and XBXXBX motif strings, the motif search was refined where B represented arginine or lysine while X lacked any acidic amino acid residues as these residues would typically repel electrostatic interactions with negatively charged GAGs [19, 20]. A clear correlation was observed between heparin‐binding M proteins and the occurrence of a XBXBX motif compared to non‐heparin‐binding M proteins (Fig. 4B). All refined XBXBX motifs were found to be distributed in the hypervariable to variable region of M proteins, corresponding to the SPR findings of M1 and M53 protein fragments described above.

To investigate whether the XBXBX motifs were surface‐exposed in M proteins to facilitate potential heparin interactions, M protein sequences were arranged into heptad registers using a Marcoil algorithm. All heparin‐binding M proteins contained at least one surface‐exposed XBXBX motif where the basic residues were not buried in the hydrophobic core (Fig. 4C).

To validate a role for this motif in M protein‐mediated heparin interactions, M53 protein was selected for site‐directed mutagenesis using alanine substitution based on previous studies [21, 22, 23, 24, 25]. Specific residues in the XBXBX heparin‐binding motif present in M53 protein (QRLKN) were targeted for alanine substitution to maintain the structural integrity of the M protein, generating a total of four site‐directed mutants for this motif (Fig. 4D). His‐tagged recombinant site‐directed mutant M53 proteins were expressed in Top10 *Escherichia coli* and purified using glutathione‐affinity chromatography coupled with Ni^2+^ − NTA chromatography (Fig. S9A,B). SDS/PAGE analysis revealed a band at 48 kDa consistent with the expected monomeric size of recombinant M53 protein (Fig. 4E) which was confirmed to be representative of M protein using α‐PAM sera (Fig. 4E). Similar to M53 protein, the appearance of doublet bands for the site‐directed mutants is characteristic of recombinant M proteins in the *E. coli* expression system [26].

To assess the contribution of XBXBX motifs in M53 protein recognition of heparin, a fluorescent plate‐based assay was conducted as previously described [13], where wild‐type M53 protein and site‐directed mutants M53^R67A^, M53^K69A^, M53^R67A,K69A^ and M53^Q66A,R67A,K69A^ were incubated with fluorescein‐labelled heparin. Binding of heparin to all site‐directed mutants resulted in large response variability, but these differences were not statistically significant compared to wild‐type M53 protein (*P* = 0.9815) (Fig. 4F). Given the complete motif sequence was not mutated, further experiments are required to definitively exclude a role for the XBXBX motif in heparin recognition by M53 protein. Nevertheless, this variable response strengthens the idea that accessory or redundant binding mechanisms along the complete M protein structure may facilitate heparin binding, coinciding with the SPR findings described for M53 proteins above.

### 5448 GAS binds GAGs via M proteins

**Fig. 5:**
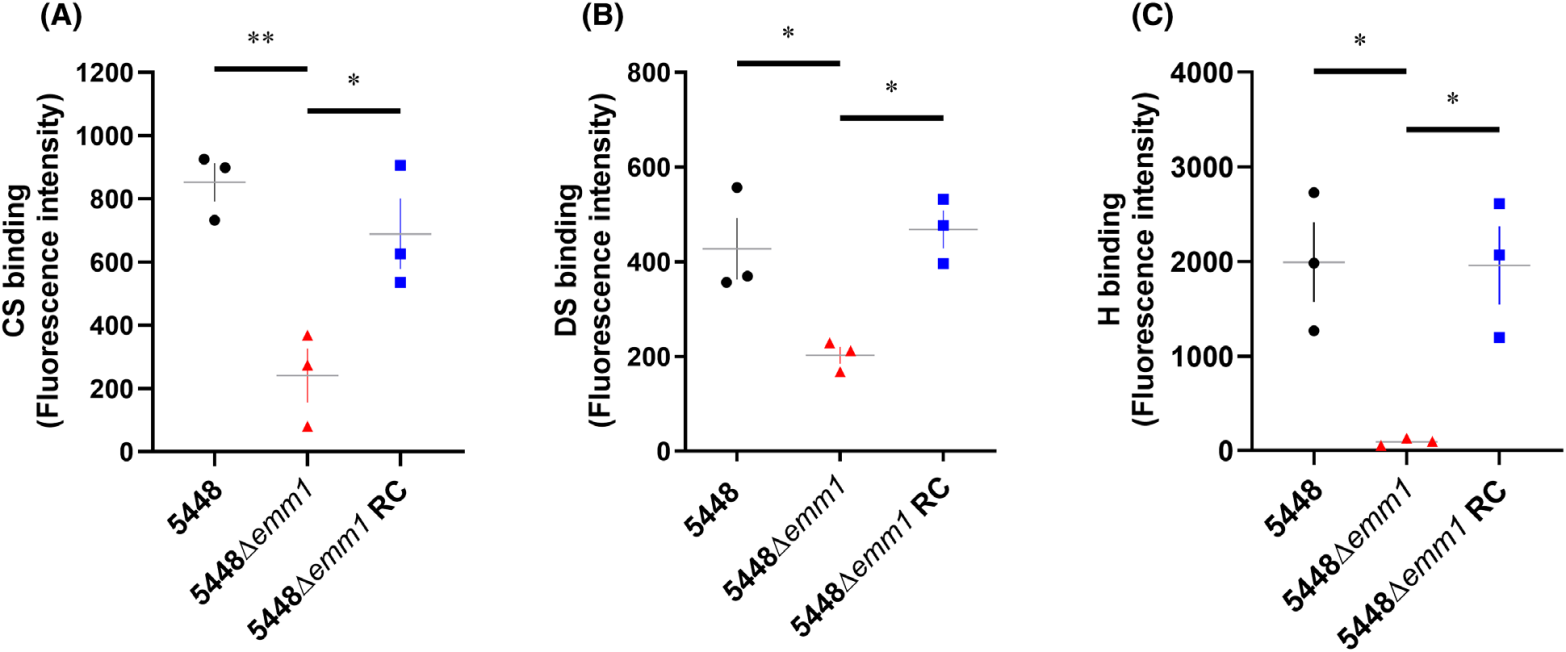
5448 GAS binds GAGs via M proteins. (A–C) Wild‐type and mutant 5448‐derived GAS strains were pre‐treated in the absence or presence of unlabelled glycosaminoglycan (GAG) in 5–10‐fold excess followed by (A) chondroitin sulfate (CS)‐fluorescein (200 μg·mL^−1^), (B) dermatan sulfate (DS)‐fluorescein (200 μg·mL^−1^) or (C) heparin (H)‐fluorescein (100 μg·mL^−1^) and analysed via geometric mean fluorescence intensity. Specific GAS–GAG interactions are shown. RC, reverse complement. (A–C) Data shown are mean ± SEM from three independent experiments. *P < 0.05 and **P < 0.01 compared to corresponding samples (one‐way analysis of variance).

It has been previously shown that M1‐, M5‐ and M6‐expressing GAS (AP1, Manfredo, and JRS4 respectively) can bind dermatan sulfate in an M protein‐dependent manner [9], but no additional studies have been conducted using other GAS strains or additional host GAGs. To investigate whether whole cell GAS can interact with a range of GAG subclasses, 5448 (A‐C pattern, M1‐expressing GAS) was selected for assessment using fluorescein‐conjugated GAGs and flow cytometry. Wild‐type 5448 was shown to bind chondroitin sulfate‐fluorescein (Fig. 5A). Deletion of the *emm* gene encoding M protein, 5448Δ*emm1*, significantly reduced fluorescence following incubation with chondroitin sulfate‐fluorescein compared to wild‐type 5448 (*P* < 0.01) (Fig. 5A). These data suggest the interaction between GAS and chondroitin sulfate is partially dependent on M protein expression. 5448Δ*emm1* reverse complement (RC) restored fluorescence intensity to that of the corresponding wild‐type 5448 (*P* = 0.5597) following incubation with chondroitin sulfate‐fluorescein (Fig. 5A). Together, the data suggest that GAS can recruit chondroitin sulfate to the cell surface in an M protein‐dependent manner.

Next, wild‐type 5448 was also shown to bind dermatan sulfate‐fluorescein. 5448Δ*emm1* displayed significantly reduced fluorescence following incubation with dermatan sulfate‐fluorescein compared to wild‐type 5448 (*P* < 0.05) (Fig. 5B) indicating the interaction between M1‐expressing GAS and dermatan sulfate is partially dependent on M protein expression as indicated previously [9]. 5448Δ*emm1* RC restored fluorescence intensity to that of the corresponding wild‐type 5448 (*P* = 0.9068) following incubation with dermatan sulfate‐fluorescein (Fig. 5B). Collectively, the data suggest that GAS can recruit dermatan sulfate to the cell surface in an M protein‐dependent manner, aligning with previous reports [9].

Finally, binding of heparin‐fluorescein to whole cell GAS was examined. Wild‐type 5448 was shown to bind heparin‐fluorescein (Fig. 5C). 5448Δ*emm1* near‐completely reduced fluorescence following incubation with heparin‐fluorescein compared to wild‐type 5448 (*P* < 0.05). These data suggest that the interaction between GAS and heparin is partially mediated by M proteins. 5448Δ*emm1* RC restored fluorescence intensity to that of the corresponding wild‐type 5448 (*P* = 0.9998) following incubation with heparin‐fluorescein (Fig. 5C). Together, the data suggest that GAS can recruit heparin to the cell surface in an M protein‐dependent manner.

### M1 proteins increase 5448 GAS adherence in the presence of chondroitin sulfate

**Fig. 6:**
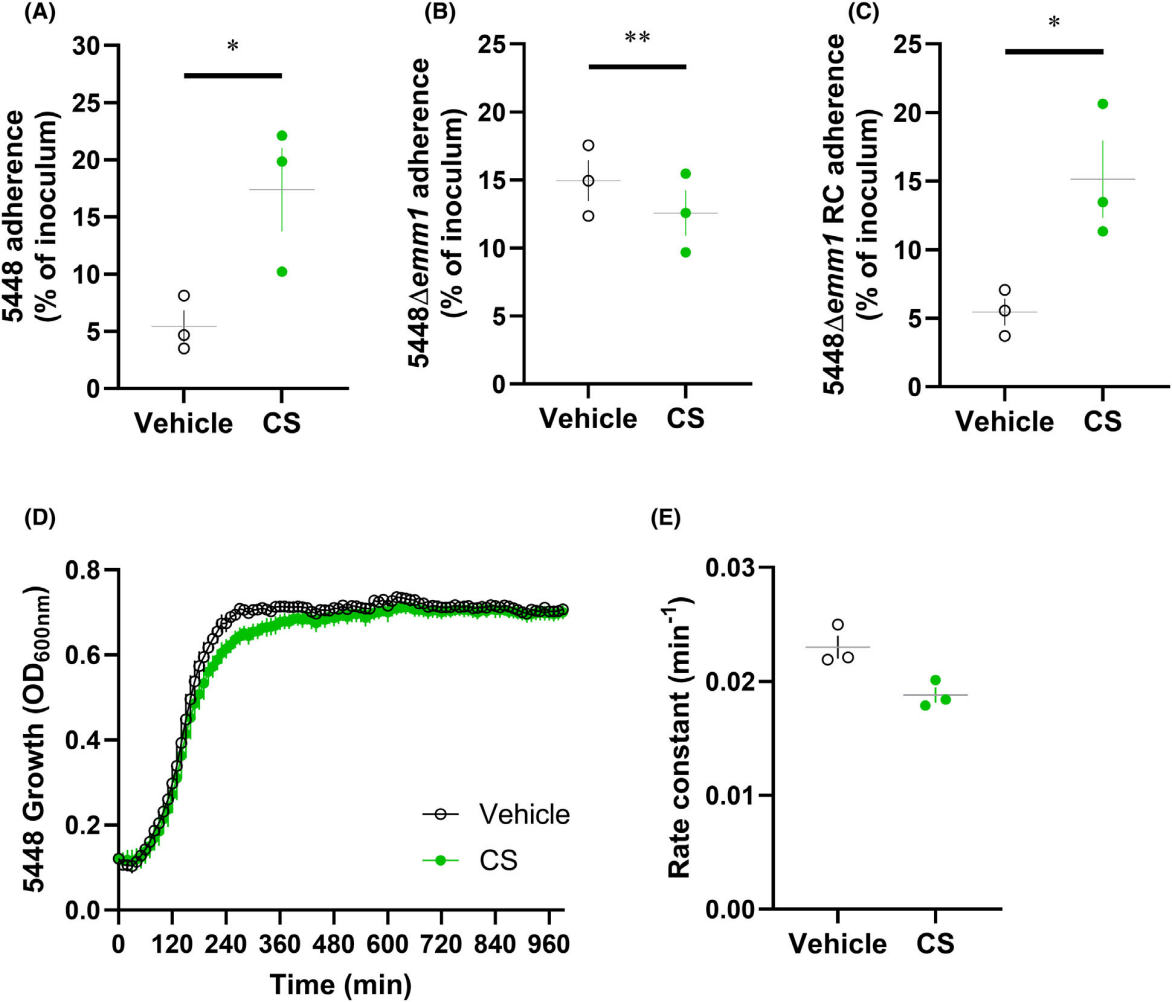
M1 proteins increase 5448 GAS adherence to HaCaT keratinocytes in the presence of chondroitin sulfate. (A) Wild‐type and (B, C) mutant 5448‐derived GAS strains were pre‐incubated with chondroitin sulfate (CS; 850 μm) or corresponding vehicle prior to infection with HaCaT keratinocytes at a MOI of 5 : 1 GAS : HaCaT. Bacterial adherence (%) was determined by enumeration of recovered bacteria relative to the original inoculum. RC, reverse complement. (D) Wild‐type 5448 GAS strains were grown in the presence of chondroitin sulfate (CS; 850 μm) or corresponding vehicle and monitored over 16‐h using spectrophotometry. (E) Rate constants were generated by non‐linear fitting of respective bacterial growth curves. (A–E) Data shown are mean ± SEM from three independent experiments. *P < 0.05 and **P < 0.01 compared to corresponding control (Student's *t*‐test).

GAGs have been previously implicated as mediators of GAS adherence to host cells [9, 10]. Since GAGs are enriched in the skin [27, 28], and GAS can adhere to keratinocytes [29] via M proteins [30], a human keratinocyte cell line (HaCaT) was selected to examine the potential of M protein–GAG interactions to modulate GAS adhesion. Pre‐incubation of wild‐type 5448 with chondroitin sulfate resulted in a significant increase in bacterial adherence to HaCaT keratinocytes compared to wild‐type 5448 pre‐incubated with vehicle (*P* < 0.05) (Fig. 6A). Pre‐incubation of 5448Δ*emm1* with chondroitin sulfate resulted in a significant decrease in bacterial adherence to HaCaT keratinocytes compared to 5448Δ*emm1* pre‐incubated with vehicle (*P* < 0.01) (Fig. 6B). This suggests that M1 protein–chondroitin sulfate interactions enhance GAS adherence to HaCaT keratinocytes, and in the absence of M protein, other surface receptors of GAS may interact with exogenous chondroitin sulfate to block adherence to host cells. The effects of pre‐incubating 5448Δ*emm1* RC with chondroitin sulfate corresponded to the wild‐type 5448 phenotype as shown by the significant increase in bacterial adherence to HaCaT keratinocytes compared to 5448Δ*emm1* RC pre‐incubated with vehicle (*P* < 0.05) (Fig. 6C). To confirm that chondroitin sulfate was not affecting bacterial growth over the course of HaCaT keratinocyte infection, bacterial growth kinetics in the presence of this GAG was assessed. Chondroitin sulfate had no effect on wild‐type 5448 growth compared to corresponding GAS incubated with vehicle, with no significant difference in growth rate (*P* = 0.0875) (Fig. 6D,E). Together, these data suggest that chondroitin sulfate increases 5448 GAS adherence to HaCaT keratinocytes via M protein‐mediated interactions.

### M1 proteins increase 5448 GAS adherence in the presence of dermatan sulfate

**Fig. 7:**
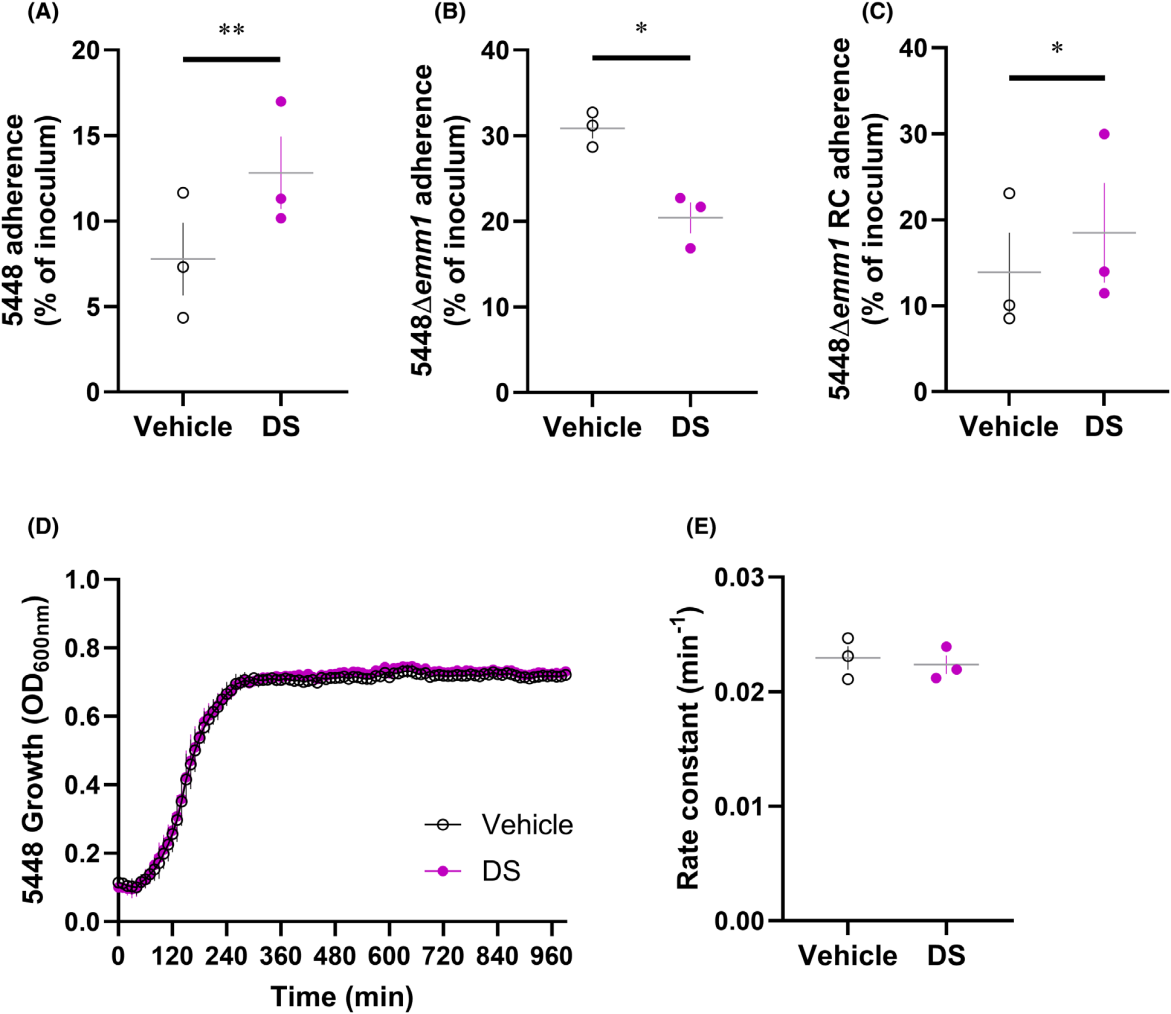
M1 proteins increase 5448 GAS adherence to HaCaT keratinocytes in the presence of dermatan sulfate. (A) Wild‐type and (B, C) mutant 5448‐derived GAS strains were pre‐incubated with dermatan sulfate (DS; 20 μm) or corresponding vehicle prior to infection with HaCaT keratinocytes at a MOI of 5 : 1 GAS:HaCaT. Bacterial adherence (%) was determined by enumeration of recovered bacteria relative to the original inoculum. RC, reverse complement. (D) Wild‐type 5448 GAS strains were grown in the presence of dermatan sulfate (DS; 20 μm) or corresponding vehicle and monitored over 16‐h using spectrophotometry. (E) Rate constants were generated by non‐linear fitting of respective bacterial growth curves. (A–E) Data shown are mean ± SEM from three independent experiments. *P < 0.05 and **P < 0.01 compared to corresponding control (Student's *t*‐test).

Next, the effect of dermatan sulfate on bacterial adherence was examined. Pre‐incubation of wild‐type 5448 with dermatan sulfate showed a significant increase in bacterial adherence to HaCaT keratinocytes (*P* < 0.01) (Fig. 7A) compared to wild‐type 5448 pre‐incubated with vehicle. In the presence of dermatan sulfate, 5448Δ*emm1* adherence to HaCaT keratinocytes was significantly decreased (*P* < 0.05) (Fig. 7B) compared to 5448Δ*emm1* pre‐incubated with vehicle. Similar to the interaction described for chondroitin sulfate, this suggests that M1 protein–dermatan sulfate interactions enhance GAS adherence to HaCaT keratinocytes, and in the absence of M protein, other surface receptors of GAS may interact with exogenous dermatan sulfate to preclude host cell adherence. As expected, pre‐incubation of 5448Δ*emm1* RC with dermatan sulfate resulted in a significant increase in bacterial adherence to HaCaT keratinocytes, compared to 5448Δ*emm1* RC pre‐incubated with vehicle (*P* < 0.05) (Fig. 7C). Finally, growth of wild‐type 5448 in the presence of dermatan sulfate revealed a similar kinetic profile to corresponding GAS incubated with vehicle with no significant differences in growth rate (*P* = 0.7173) (Fig. 7D,E) indicating this GAG does not modulate bacterial cell number directly. Collectively, the data indicate that dermatan sulfate increases 5448 GAS adherence to HaCaT keratinocytes via M protein‐mediated interactions.

### Heparin and heparan sulfate do not modulate 5448 GAS adherence

**Fig. 8:**
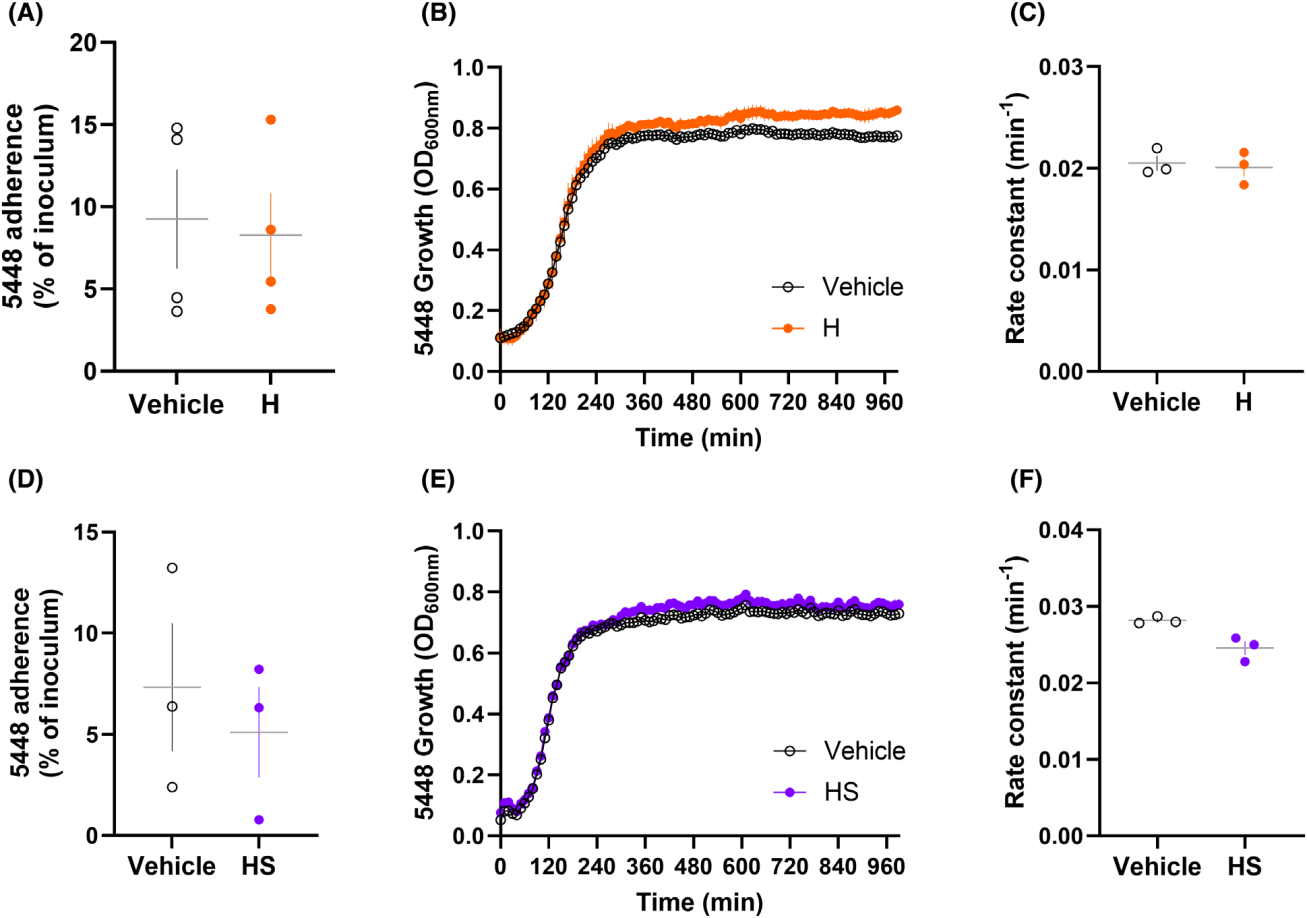
5448 GAS adherence to HaCaT keratinocytes is not modulated by heparin or heparan sulfate. (A, D) Wild‐type 5448 was pre‐incubated with (A) heparin (H; 65 μm) or (D) heparan sulfate (HS; 1 μm) or corresponding vehicle prior to infection with HaCaT keratinocytes at a MOI of 5 : 1 GAS:HaCaT. Bacterial adherence (%) was determined by enumeration of recovered bacteria relative to the original inoculum. (B, C, E, F) Wild‐type 5448 GAS strains were grown in the presence of (B, C) heparin (H; 65 μm) or (E, F) heparan sulfate (HS; 1 μm) corresponding vehicle and monitored over 16‐h using spectrophotometry. Rate constants were generated by non‐linear fitting of respective 5448 growth curves. (A–F) Data shown are mean ± SEM from (B–F) three or (A) four independent experiments. (A, C, D, F) Data were analysed using Student's *t*‐tests (*P* > 0.05).

Finally, the effect of heparin on GAS adherence was investigated. In contrast to the previous GAGs, pre‐incubation of wild‐type 5448 with heparin did not increase bacterial adherence to HaCaT keratinocytes compared to corresponding GAS pre‐incubated with vehicle (*P* = 0.2994) (Fig. 8A). 5448 growth was not affected by the presence of heparin, with no significant difference in growth rate compared to corresponding GAS incubated with vehicle (*P* = 0.6054) (Fig. 8B,C).

Since heparan sulfate did not bind M1 proteins using SPR, this GAG was utilised as a specificity control to validate the M1 protein‐dependent increase in GAS adherence to HaCaT keratinocytes from chondroitin sulfate and dermatan sulfate interactions described above. As expected, pre‐incubation of wild‐type 5448 with heparan sulfate did not significantly increase bacterial adherence to HaCaT keratinocytes compared to wild‐type 5448 pre‐incubated with vehicle (*P* = 0.3183) (Fig. 8D). Similar to all other GAGs, heparan sulfate had no significant effects on bacterial growth over time (*P* = 0.0622) (Fig. 8E,F).

## Discussion

High‐affinity binding events with proteins and protein derivatives have often been the focal point in understanding M protein‐mediated GAS adhesion, with investigations into the lectin‐binding properties of M proteins limited. The current study provides a comprehensive evaluation of M protein interactions with sulfated GAGs, with evidence of both conserved and *emm* type‐specific functions based on *emm* pattern and shared binding domains between M proteins. Novel M protein‐dependent interactions of 5448 GAS with chondroitin sulfate, dermatan sulfate and heparin and their role in bacterial adherence were described, with M1 protein‐mediated interactions with chondroitin sulfate and dermatan sulfate promoting GAS adherence to HaCaT keratinocytes.

Investigations into lectin‐like interactions are challenging due to the structural complexity of glycans. The synthesis of GAGs is dependent on enzyme expression which, in turn, can affect disaccharide composition [31]. Heterogeneity within each class of GAGs arises due to highly variable sulfation patterns or chain length which may also be altered in a disease state [32]. This diversity further obscures mechanisms driving the specificity of protein–GAG interactions. This study systematically categorised conserved glycan binding function across 11 major *emm*‐cluster groups, further evaluating the functionality of a full‐length M protein sequence‐based classification system [33]. However, ligand presentation can be affected by glycan linkages, orientation, density and size and by other environmental factors such as humidity, temperature and pH [34, 35]. As such, binding interactions detected by glycan microarrays have been previously validated by SPR [36], an analytical tool which offers label‐free analyses of real‐time binding events in a microfluidic environment. In the context of the current study, a key advantage of utilising SPR was to provide a model that better mimics both the dynamicity of soluble GAGs found in exudate [37] and surface‐anchored expression of M proteins, for the molecular characterisation of M protein–GAG interactions. M proteins representing 12 different *emm* types were included in this study. Currently, there are over 200 *emm* types globally distributed [38], and while there were clear relationships between *emm* pattern and specific GAG recognition defined by SPR analysis, a larger sample size is required to definitively conclude *emm* pattern‐specific interactions.

M proteins were found to interact with dermatan sulfate with affinities in the nanomolar range, confirming previous findings [9]. Furthermore, M proteins were shown to have high‐affinity interactions with chondroitin sulfate, heparin and heparan sulfate. For most M proteins, equilibrium dissociation constants (*K*_D_) for all GAGs concurred with previously reported *K*_D_ values of other host GAG‐binding proteins [1] and consistent with a previous report on GAG–protein interactions using SPR [39]. It is possible that M proteins may engage in multivalent GAG interactions, thus affecting the observed binding affinities in this study; however, avidity and stoichiometry were not examined. Heparin and heparan sulfate were shown to specifically interact with M proteins of A‐C and D pattern strains, which have been reported to preferentially infect the throat and skin respectively [40]. M proteins derived from E pattern strains, which are equally represented in both tissue sites, are characteristically smaller in size [41]. Therefore, a reduction in size and amino acid sequence of these M proteins may limit the available binding sites that accommodate GAG interactions. Since both M53 and M1 protein truncation mutagenesis suggested a predominant heparin‐binding site may exist within the hypervariable‐variable region of the protein as opposed to the conserved C repeat region, this supports the idea that M proteins of E pattern strains may have lost a heparin‐binding site at the N terminus as a result of evolutionary divergence in sequence. Similarly, truncation mutagenesis of M53 protein suggested a linear binding motif for heparan sulfate may exist in the variable region of the protein that is situated between the B and C repeats, a sequence that may be lost in M proteins of E pattern strains. Previous studies identifying putative heparan sulfate‐binding motifs have suggested the involvement of basic amino acid residue clusters [24, 42], particularly lysine clusters [25] which are found in this discrete region of M53 protein; however, the role for these lysine clusters in M53 protein‐mediated binding to heparan sulfate was not explored here.

SPR analysis of truncated M1 protein fragments revealed conflicting results for the mapping of domains responsible for heparin binding. Previous work on M1 proteins has suggested that domains that immediately precede the C repeat region function to stabilise the coiled‐coil tertiary structure which is essential for interactions with some ligands [43]. On the other hand, the non‐ideal destabilising residues present in the B repeats of M1 protein sequence are considered a prerequisite for other protein interactions, specifically fibrinogen [44, 45]. Moreover, the domain spanning the hypervariable region to B repeats can also facilitate the transition of monomer M1 proteins to dimers [45], which may impact protein conformations that immobilise to the SPR sensor chip surface. M protein dimerisation has been shown to enhance protein‐binding function [46, 47], but is temperature‐sensitive with stable dimer formation at 25 °C [46] which corresponds to the control temperature selected for single‐cycle kinetics of GAG interactions. Therefore, the propensity of M proteins to form dimers in solution [48, 49] coupled with the flexibility of exposed B repeats in M protein fragments may accommodate higher affinity interactions with heparin than with full‐length M1 protein. As M proteins of E pattern strains rarely harbour B repeat regions [41] as represented by M2, M9 and M60 protein in this study, none of which bound heparin, heparin‐binding appears to be dependent on the properties of the B repeat region.

Discrete M protein domains were shown to facilitate heparin interactions which led to the proposal of linear XBXBX sequence motifs. Numerous GAG binding sequences rely on ionic interactions between the negatively charged carboxylic acid groups or sulfate groups present in the GAG chains with positively charged basic residues arginine, lysine and histidine in proteins [50]. Given the isoelectric point of histidine is at neutral pH [51], and infected wound beds are alkaline [52, 53], it was hypothesised that histidine residues, which are seldom present in M protein sequences, would shift to a deprotonated state. While the presence of histidine residues in M proteins may help strengthen ionic interactions with GAGs, an essential role in GAG binding is unlikely hence these residues were omitted from binding consensus sequence searches. Since GAGs have various free hydroxyl groups, it has become clear that non‐ionic interactions such as hydrogen bonding and Van der Waals forces can significantly contribute to protein–GAG interactions [54, 55]. It is plausible that accessory interactions with polar or uncharged residues spanning the protein backbone would further reinforce GAG interactions. This is supported by the finding that while GAG binding to truncated mutants of M proteins was detected, affinities to some GAGs were reduced compared to the full‐length M proteins. Moreover, it has been previously reported that disruption of a GAG‐binding motif in a bacterial adhesin can increase the *K*_D_ by a 100‐fold [56], compared to the 2–5‐fold difference between M protein fragments interacting with GAGs observed here which further indicates a cooperative binding mechanism.

No linear GAG binding sequences on M proteins were identified for chondroitin sulfate or dermatan sulfate. This is partly due to the fact that ligands for these GAGs are less well‐characterised in the literature. However, a systematic experimental process known as GAGDoMA has been recently published, which aids the identification of potential protein‐binding sequences present on chondroitin sulfate and dermatan sulfate chains [57]. This technology implicates chain length and sulfation pattern (sites and number) as defining features of potential motifs within these two GAGs [57], an approach that could be harnessed in future studies.

There is growing evidence to suggest that protein conformation including secondary or tertiary structure, and the spatial distribution of basic amino acid residues can affect GAG interactions [58, 59, 60, 61, 62]. The proposed heparin‐binding XBXBX motif present in the hypervariable to variable region of M proteins is evidence of sequence diversity that retains functionality, indicating a larger role for conformational motifs in GAG interactions and is further supported by our site‐directed mutagenesis findings. This has been reported for other M protein‐mediated interactions [63]. Several studies have observed conformational binding patterns for heparin recognition [64, 65], including one study which identified a structural CPC clip motif signature based on the spatial distances between two cationic residues and one polar residue of the protein [66]. Heptad registers defining the M protein can shift upon interaction with specific ligands, due to the instability of the M protein central region [67]; hence, it is difficult to predict conformational motifs defining M protein–GAG interactions.

Although heparin and heparan sulfate are GAGs that are often reported interchangeably or grouped in the literature, their structural compositions are distinct [1] with functional differences in protein binding capabilities [68, 69]. This may underpin the differences observed in the recognition of these two GAGs by M proteins. Heparan sulfate was shown to interact with only two M proteins a high affinity, in contrast to heparin which interacted with seven of the 12 M proteins. One key physiochemical difference between heparin and heparan sulfate centres on sulfation pattern. While both GAGs comprise an identical number of potential sites that can be sulfated [1, 70], heparin averages ~ 2.7 sulfate groups per disaccharide while heparan sulfate contains ≤ 1 sulfate groups per disaccharide [71]. This provides heparin with a greater negatively charged surface density than heparan sulfate or other GAGs [72]. This may facilitate stronger interactions with M proteins via increased ionic interactions. The major configuration of heparin disaccharides comprises L‐IdoA, which provides structural flexibility to the GAG structure [73], and, in turn, can promote changes in the spatial positioning of sulfate groups to present more favourable binding interactions between heparin and proteins [74]. In addition to differences in structural dynamics influencing binding interactions of heparin and heparan sulfate, the distribution of these GAGs in human tissue may offer functional insight into differences in M protein specificity. Heparan sulfate is expressed on the surface by almost all cell types and found in the ECM and basement membranes; however, the high level of structural heterogeneity that exists within heparan sulfate chains compared to other GAGs may explain why binding to select M proteins occurred but was not a conserved function across all M proteins [75]. In contrast, heparin is abundant in, and secreted by, mast cells of the circulatory system [76, 77] with anti‐inflammatory and anti‐coagulant functions [78, 79]. However, both heparin and heparan sulfate are implicated in wound repair processes [32, 80]. Since A‐C pattern strains have a propensity to cause invasive infection in sites of deep tissue and vasculature [40, 81], and D pattern strains are predominately associated with localised skin infections such as impetigo [82], tissue specificity may drive strain‐specific interactions with heparin and heparan sulfate that is mediated by M proteins to disrupt host homeostatic processes.

Whole cell analysis of GAS–GAG interactions demonstrated that 5448 GAS could bind dermatan sulfate via M proteins, corresponding to earlier findings [9]. It was also suggested that heparin and heparan sulfate interact with GAS via a shared receptor such as M proteins, which was indirectly supported through whole cell inhibitory binding studies using radiolabelled dermatan sulfate [9]. The current study confirms for the first time that GAS can interact with other GAG subclasses including heparin in an M protein‐dependent manner. Although only 5448 GAS was included in this study, it is possible that strain‐specific interactions with GAGs may occur, governed by differences in M protein receptor availability or distribution on the GAS cell surface [13], or alternatively due to variations in GAG source, chain length, sulfation pattern and/or purity [1, 83]. Whole cell GAS interactions with GAGs may also be affected by other factors including bacterial surface electrostatic charge as well as neighbouring receptors which could either facilitate or physically hinder potential GAG interactions [84]. It has also been previously shown that GAS‐secreted proteases, presumably SpeB, can actively liberate GAGs such as dermatan sulfate from proteoglycans *in vitro* [7], and it is known that soluble GAGs are abundant in wound microenvironments [37]. Therefore, through the acquisition of circulating dermatan sulfate or other released GAGs from host cell or bacterial‐induced ECM damage, this may represent a potential novel mode of GAS adherence to keratinocytes and other cell types.

GAS typically colonise epithelial tissue of the skin or throat [85], and the role of the M protein in cell adhesion is dependent upon specific *emm* type, GAS protease expression, the degree of strain encapsulation, stage of infection, host cell specificity and tissue tropism [30, 86]. It has also been shown that GAS can adhere to keratinocytes in an inoculum‐dependent manner indicative of a receptor‐mediated process [87]. Coinciding with this idea of a receptor‐mediated process, the recruitment of chondroitin sulfate and dermatan sulfate by M1 proteins of 5448 GAS was shown to increase bacterial adherence to HaCaT keratinocytes. Thus, this may be indicative of an adhesive bridging mechanism [88] via a linker protein present in the ECM or on the host cell involved in GAG recognition. Collagen IV is highly concentrated in the basement membrane, an ECM between the skin epidermis and dermis [89] and is synthesised by HaCaT keratinocytes [90]. Moreover, collagen IV is a recognised binding receptor of endogenous chondroitin sulfate and dermatan sulfate [91, 92] suggesting collagen IV may be the linker protein facilitating GAS adherence in this study. Although heparin can also bind collagen IV [92], chondroitin sulfate and dermatan sulfate are structurally similar with a shared GalNAc linkage that is exclusive to these two GAGs from the GAG subclasses, which may underlie a putative binding site with collagen IV or other host proteins.

In conclusion, this work has determined that different M proteins of GAS can bind to a variety of sulfated host GAGs with high affinity, with evidence of M protein‐mediated interactions with chondroitin sulfate and dermatan sulfate increasing GAS adherence to human skin keratinocytes. A prerequisite for persisting GAS infection is efficient adhesion to host cells for colonisation and subsequent evasion of the host immune response, and this work serves to highlight GAGs as an attractive therapeutic target for the clearance of GAS infection.

## Materials and methods

### Glycosaminoglycans (GAGs)

Chondroitin sulfate (A/C) sodium salt (63%/37%; Cat# C8529; 20 kDa), chondroitin sulfate (B)/dermatan sulfate sodium salt (Cat# C3788; 25 kDa), heparan sulfate sodium salt (Cat# H7640; 50 kDa) and heparin sodium salt (Cat# H3149; 15 kDa) were from Sigma‐Aldrich (St Louis, MO, USA). Fluorescein‐conjugated heparin (Cat# HP‐201; 27 kDa) was from Creative PEGworks (Chapel Hill, NC, USA), while fluorescein‐conjugated chondroitin sulfate (A/C) and dermatan sulfate were generated by Creative PEGworks using the respective non‐fluorescent GAGs sourced above. Epimer configuration was not disclosed by suppliers.

### GAS culture

All GAS strains have been described previously [13]. Briefly, the isogenic *emm1*‐deletion mutant was replaced with a non‐expressing chloramphenicol resistance gene using a temperature‐sensitive pHY304 vector. Restoration of the *emm1* gene was conducted using a pDCerm vector containing an erythromycin resistance gene [93]. GAS strains were cultured under static conditions at 37 °C in 3% (w/v) Todd Hewitt broth (BD Biosciences, Franklin Lakes, NJ, USA) with 1% (w/v) yeast (Sigma‐Aldrich). Cultures were grown in absence or presence of 5 μg·mL^−1^ erythromycin until mid‐logarithmic phase as described previously [13].

### Mammalian cell culture

HaCaT human keratinocytes (CLS Cat# 300493/p800_HaCaT, RRID:CVCL_0038) [94] were maintained in Dulbecco's modified Eagle medium: Nutrient Mixture F‐12 (DMEM/F12; Thermo Fisher Scientific, Waltham, MA, USA) containing heat‐inactivated 10% (v/v) foetal bovine serum (Bovogen Biologicals, Melbourne, Vic., Australia), 2 mm GlutaMAX, 100 U·mL^−1^ penicillin and 100 μg·mL^−1^ streptomycin (Thermo Fisher Scientific) at 37 °C/5% CO_2_. HaCaT cells were authenticated by short tandem repeat profiling (Garvan Institute of Medical Research, Sydney, NSW, Australia) and were routinely shown to be negative for mycobacterial contamination using the MycoAlert Mycoplasma Detection Kit (Lonza, Basel, Switzerland) as per manufacturer's instructions.

### Recombinant M protein and M protein fragment expression and purification

Full‐length and truncated M protein design and expression has been described previously [11, 13]. Similarly, site‐directed mutant M53 proteins were designed into a pGEX‐2T vector containing a glutathione‐S‐transferase tag to facilitate purification. Briefly, expression of recombinant M proteins (including site‐directed mutants) and M protein fragments, all integrated with a C‐terminal His‐tag, was induced using 0.1 m isopropyl β‐D‐1‐thiogalactopyranoside over 4‐h in BL21(DE3) or Top10 *E. coli* (Thermo Fisher Scientific). Clarified lysates were purified using glutathione‐affinity chromatography (recombinant M proteins including site‐directed mutants) and/or Ni^2+^‐NTA affinity chromatography (recombinant M proteins including site‐directed mutants and M protein fragments) and/or ion‐exchange chromatography (M protein fragments).

### Polyacrylamide gel electrophoresis and immunoblotting

Concentrated purified recombinant M proteins (including site‐directed mutants) and M protein fragments (2 μg) were electrophoresed using 10–12% polyacrylamide under reducing conditions and visualised using Coomassie R‐250 Rapid staining [95] or Colloidal Blue staining [96]. Immunoblotting of M protein and M protein fragments (200 ng) was conducted using rabbit polyclonal anti‐M protein sera (1 : 30 000), as described previously [13], with bovine serum albumin included as a negative control.

### Glycan microarray

Recombinant M protein–glycan interactions were screened using a glycan microarray as previously described [11, 13]. Briefly, recombinant M proteins (0.5–1 μg) were complexed with mouse anti‐histidine IgG (Cat# 2366; Cell Signaling Technology), rabbit anti‐mouse IgG Alexafluor488 conjugate (Cat # A‐11059; Thermo Fisher Scientific) and goat anti‐rabbit IgG Alexafluor488 conjugate (Cat# A‐11008; Thermo Fisher Scientific) at a ratio of 4 : 2 : 1 and immobilised onto pre‐blocked glycan microarray slides. Fluorescence was detected using a ProScanArray Microarray 4‐Laser Scanner (Perkin Elmer, Waltham, MA, USA). An overview of the MIRAGE compliant method for the production and quality control of the glycan array as well as the complete list of glycan structures utilised in this study is provided (Tables S1 and S2).

### Surface plasmon resonance (SPR)

M protein–GAG binding interactions were monitored in real time using surface plasmon resonance, as previously described [13]. Briefly, recombinant His‐tagged M proteins (200 nm) were immobilised to a Series S NTA Sensor Chip (Cytiva, Marlborough, MA, USA) and GAGs (0–200 nm) were sequentially injected with a 180‐s association time and a 30‐s dissociation time. Streptokinase (in‐house) was used as a universal negative control as it did not bind any of the M proteins tested. Human serum albumin (Cat# A3782; Sigma‐Aldrich), fibrinogen (Cat# F4883l; Sigma‐Aldrich), glu‐plasminogen (Cat# HCPG‐0130; Prolytix, Essex Junction, VT) and C4b‐binding protein (Cat# 600672; Agilent Technologies, Santa Clara, CA, USA) were included as positive controls for specific M proteins [33]. Interactions were evaluated using steady‐state affinity analysis which followed a 1 : 1 Langmuir binding model [97].

### Fluorescent solid‐phase heparin‐binding assay

A fluorescent heparin‐binding assay was performed as previously described [13]. Briefly, wild‐type and site‐directed mutant M53 proteins (20 μg·mL^−1^), or bovine serum albumin as a negative control, were immobilised to black‐walled μClear® bottom plates (Greiner Bio‐One; Kremsmünster, Austria) and incubated with fluorescein‐labelled heparin (100 μg·mL^−1^). Fluorescence in the presence of pre‐incubated unlabelled GAG (30‐fold excess) was used as a proxy for non‐specific binding. Recordings were measured using a CLARIOstar plate reader. Wild‐type M53 proteins were also incubated with anti‐human CXCR3 fluorescein‐conjugated antibody (Cat# FAB160F; R&D Systems, Minneapolis, MN, USA) or mouse immunoglobulin G FITC‐conjugated antibody (Cat# 555748; BD Biosciences) as negative controls to confirm heparin binding of M proteins was not mediated by fluorescein.

### Flow cytometry

Mid‐logarithmic GAS strains (1 × 10^6^ cells) were pre‐incubated in the absence or presence of unlabelled GAG in excess (1–1.5 mg·mL^−1^) on ice for 1 h. Cells were washed with phosphate‐buffered saline and incubated with 100–200 μg·mL^−1^ fluorescein‐labelled GAG (as indicated) and 10 μg·mL^−1^ cell viability dye 7‐aminoactinomycin D (Cayman Chemical, Ann Arbor, MI, USA) for 1 h on ice protected from light. Labelled cells were collected and analysed by flow cytometry as previously described [13].

### GAS adherence assay

A keratinocyte cell adherence assay was conducted as previously described [13]. Briefly, mid‐logarithmic GAS (2.5 × 10^5^ cells) were pre‐incubated with 1 μm – 0.85 mm GAG (as indicated; or corresponding vehicle) for 30‐min at room temperature and added to HaCaT keratinocyte monolayers at a MOI of 5 : 1 for 1‐h at 37 °C. To assess total bacterial cell association (adherence and invasion), GAS‐bound HaCaT keratinocytes were detached using 500 U·mL^−1^ accutase (Invitrogen, Waltham, MA, USA) with a 10‐min incubation at 37 °C followed by 0.025% (v/v) Triton® X‐100 for cell lysis. Well selection for medium extraction was randomised to minimise count bias. A 10‐fold serial dilution was performed, and spot plated for bacterial enumeration. To measure GAS invasion only, GAS‐bound HaCaT keratinocytes were pre‐treated with 100 μg·mL^−1^ gentamicin sulfate as described previously [11]. Bacterial counts (CFU) were normalised to the original inoculum. The level of GAS cell adherence was determined by subtracting the percentage (%) of internalised GAS from the percentage (%) of total associated GAS.

### Bioinformatic analyses for GAG motif processing

Searches for established linear heparin‐binding motifs [18] on M protein sequences [13] were performed manually, and frequency was recorded. Putative consensus sequences on M proteins were refined based on biochemical properties of the primary amino acid sequence. This included, but was not limited to, ionic charge, hydrophobicity, acidity and alkalinity that would promote or restrict the interaction, but also accounting for the environmental conditions of the physiological environment where the proposed interaction would likely occur (e.g. pH). *In silico* modelling of M protein heptad registers was conducted using Waggawagga [98], a coiled‐coil prediction tool paired with a Marcoil algorithm [99].

### Statistical analyses

Data are presented as mean ± standard error of the mean (SEM) of at least three independent experiments. All independent experiments were conducted using three technical replicates. Where applicable, plate structure was routinely altered to mitigate positioning bias within data sets. Curve fitting and statistical analyses were conducted using graphpad prism 8.4.2 (GraphPad Software, La Jolla, CA, USA). Rate constants (*k*) for bacterial growth were generated by a non‐linear regression model using the logistic growth equation with least squares fit. All data presented were normally distributed (Shapiro–Wilk). A Student's *t*‐test, with Welch's correction where appropriate, was performed for single comparisons, while a one‐way analysis of variance with Sidak's *post hoc* test was used to determine significant differences for multiple comparisons. *P* < 0.05 was considered significant. Non‐significant differences are not shown. Statistical analysis of glycan microarray data has been outlined previously [11].

## Conflict of interest

The authors declare no conflict of interest.

## Author contributions

All authors have read and approved the final manuscript. TB‐DM (Conceptualisation, investigation, methodology, formal analysis, writing—original draft, visualisation); DMPDO (Investigation, formal analysis, methodology, resources); EKS (Investigation, formal analysis); LEH‐T (Investigation, methodology); CJD (Investigation, methodology); MJW (Conceptualisation, resources); MPJ (Conceptualisation, funding acquisition, methodology, resources); RS (Conceptualisation, methodology, validation, supervision, resources, writing—review and editing); MLS‐S (Conceptualisation, funding acquisition, methodology, validation, project administration, supervision, resources, writing—review and editing).

## Supporting information


**Fig. S1.** Steady‐state affinity curves of M protein–chondroitin sulfate interactions.
**Fig. S2.** Steady‐state affinity curves of M protein–dermatan sulfate interactions.
**Fig. S3.** Steady‐state affinity curves of M protein–heparin interactions.
**Fig. S4.** Steady‐state affinity curves of M protein–heparan sulfate interactions.
**Fig. S5.** Steady‐state affinity curves of M1 protein fragment–chondroitin sulfate interactions.
**Fig. S6.** Steady‐state affinity curves of M53 protein fragment – heparan sulfate interactions.
**Fig. S7.** Steady‐state affinity curves of M protein fragment–dermatan sulfate interactions.
**Fig. S8.** Steady‐state affinity curves of M protein fragment–heparin interactions.
**Fig. S9.** Expression and purification profile of site‐directed mutant M53 proteins.
**Table S1.** Glycan microarray information as per MIRAGE guidelines
**Table S2.** Glycan structures present on the microarray.

## Data Availability

The data that support the findings of this study are available on request from the corresponding author.
