# Supplementary material for: Group A Streptococcus interacts with glycosaminoglycans via M proteins to modulate bacterial adherence in vitro
Source: FEBS J. 2025 Jul 3;292(20):5540–62. doi: 10.1111/febs.70167 (PMC12524986; doi:10.1111/febs.70167)
Supplement: Supplementary file 1 — Fig. S1. Steady‐state affinity curves of M protein–chondroitin sulfate interactions. Fig. S2. Steady‐state affinity curves of M protein–dermatan sulfate interactions. Fig. S3. Steady‐state affinity curves of M protein–heparin interactions. Fig. S4. Steady‐state affinity curves of M protein–heparan sulfate interactions. Fig. S5. Steady‐state affinity curves of M1 protein fragment–chondroitin sulfate interactions. Fig. S6. Steady‐state affinity curves of M53 protein fragment – heparan sulfate interactions. Fig. S7. Steady‐state affinity curves of M protein fragment–dermatan sulfate interactions. Fig. S8. Steady‐state affinity curves of M protein fragment–heparin interactions. Fig. S9. Expression and purification profile of site‐directed mutant M53 proteins. Table S1. Glycan microarray information as per MIRAGE guidelines Table S2. Glycan structures present on the microarray. [file FEBS-292-5540-s001.pdf]

Table S1: Glycan microarray information as per MIRAGE guidelines

Classification	Guidelines
**1. Sample: Glycan binding sample**	
Description of sample	<u>Sample/Origin:</u><br>M proteins of *Streptococcus pyogenes*; produced as recombinant proteins in Top10 *Escherichia coli*.<br><u>Method of preparation:</u><br>Preparation of recombinant M proteins is as described in the Materials and Methods section. Densitometric analysis revealed protein purity to be range from 95-100%.
Sample modifications	All recombinant M proteins expressed a C-terminal His-tag.
Assay protocol	As described in the Materials and Methods section.
**2. Glycan Library**	
Glycan description for defined glycans	Glycans used in this study are outlined in Supplementary Table S2 and is a published library in doi: 10.1371/journal.pntd.0004120.
Glycan description for undefined glycans	N/A.
Glycan modifications	Glycans (with IDs in number only format) were obtained from Prof. Nicolai Bovin and were modified with spacers as per DOI: 10.1073/pnas.0407902101. The library of these glycans was first published in DOI: 10.1016/j.molimm.2009.06.010.
**3. Printing surface (Microarray Slide)**	
Description of surface	Epoxy activated glass microarray slides.
Manufacturer	ArrayIt SuperEpoxy 3 (SME3).
Custom preparation of surface	N/A.
Non-covalent Immobilisation	N/A.
**4. Arrayer (Printer)**	
Description of Arrayer	SpotBot® Extreme Protein Microarray Spotter (ArrayIt, California, USA).
Dispensing mechanism	Contact printing using 946NS6 pins with a 6 pin in a 3 columns x 2 rows configuration.
Glycan deposition	Approximately 1.8 nL per spot is printed according to manufactures guidelines.<br>Glycans were at 500 μM in 50:50 DMF:DMSO.

Printing conditions	Arrays were printed with dehumidification at a maximum humidity of 60% relative humidity (Standard laboratory starting humidity of 75-90%) at 22ºC. Glycans were left to react with the slide for at least 8 hours after the print was completed.
**5. Glycan Microarray with "Map"**	
Array layout	The array consists of a single array of glycans split between 6 pins (3 columns x 2 rows) with 4500 µm row and column spacing. Each pin printed a 20 columns x 16 rows with 200 µm spot spacing (centre to centre) with a minimum spot size of 100 µm. Each sample is printed in quadruplicate with each of the 6 print areas including at least three negative control samples (print solution only) and two positive control samples consisting of one sample of fluoresceinamine and one sample of a mixture of rabbit anti-mouse antibody labelled with Alexa 555 and Alexa 647. Positive controls provide proof of successful immobilization of the amine reagents and provides for orientation for analysis. The antibodies can also provide controls for secondary antibodies used in experiments (if applicable).
Glycan identification and quality control	Arrays are quality controlled by a range of measures. 1. Each printed array is post print scanned to confirm deposition of the glycans on the array surface prior to neutralization of the remaining slide surface. 2. Post neutralized slides are scanned again to monitor for remaining autofluorescence. 3. Slides are assayed with fluorescently labeled lectins: WGA-Texas Red (EY Laboratories) and ConA-FITC (EY Laboratories).
**6. Detector and Data Processing**	
Scanning hardware	ProScanArray 4 laser (488 nM, 532 nM, 595 nM, 647 nM) scanner (Perkin Elmer).
Scanner settings	Scanning resolution: 10 µM.<br>Laser channel: 595nM excitation / 625nM emission filter.<br>PMT: 70% gain.<br>Scan powers: 100% laser power.
Image analysis software	ScanArray Express (Perkin Elmer).
Data processing	Data was exported as a CSV file and exported to Microsoft Excel.
**7. Glycan Microarray Data Presentation**	
Data presentation	Data is presented as a heat map indicating yes/no binding of M protein clusters in Figure 1. Data including glycan identification are presented in Table S2. Raw and/or analysed values of binding are available upon request.
**8. Interpretation and Conclusion from Microarray Data**	
Data interpretation	We only use glycan arrays as a yes/no binding tool. Due to this we look only at binding that is unambiguously above background vs. lack of binding above background. Average background + 3 times the standard deviation of the background of 20 sets of 4 spots of DMF:DMSO only spots is applied to determine if binding observed is significantly above background. Only spots with values equal to or greater than this

	value were considered as binding from data of any tested slide. These values are slide dependent.
Conclusion	Phylogenetically diverse M proteins have broad specificity for glycan recognition.

Table S2: Glycan structures present on the microarray.

	Glycan index	Structure
Terminal galactose	1	Galα
	2	Galβ
	3	Galα1-2Galβ
	4	Galα1-3Galβ
	5	Galα1-3GalNAcβ
	6	Galα1-3GalNAcα
	7	Galα1-3GlcNAcβ
	8	Galα1-4GlcNAcβ
	9	Galα1-6Glcβ
	10	Galβ1-2Galβ
	11	Galβ1-3GlcNaAcβ
	12	Galβ1-3Galβ
	13	Galβ1-3GalNAcβ
	14	Galβ1-3GalNAcα
	15	Galβ1-4Glcβ
	16	GalNAcα1-3GalNAcβ
	17	GalNAcα1-3Galβ
	18	GalNAcα1-3GalNAcα
	19	GalNAcβ1-4GlcNAcβ
	20	Galβ1-2Galα1-4GlcNAcβ
	21	Galβ1-3Galβ1-4GlcNAcβ
	22	Galβ1-4GlcNAcβ1-3GalNAcα
	23	Galβ1-4GlcNAcβ1-6GalNAcα
	24	GalNAcβ1-4Galβ1-4Glcβ
	25	Galβ1-3GalNAcβ1-3Gal
	26	Galβ1-4Galβ1-4GlcNAc
	27	Galα1-3Galβ1-4GlcNAcβ1-3Galβ
	28	Galα1-4GlcNAcβ1-3Galβ1-4GlcNAcβ
	29	Galβ1-3GlcNAcβ1-3Galβ1-3GlcNAcβ
	30	Galβ1-3GlcNAcα1-3Galβ1-4GlcNAcβ
	31	Galβ1-3GlcNAcβ1-3Galβ1-4GlcNAcβ
	32	Galβ1-3GlcNAcα1-6Galβ1-4GlcNAcβ
	33	Galβ1-3GlcNAcβ1-6Galβ1-4GlcNAcβ
	34	Galβ1-3GalNAcβ1-4Galβ1-4Glcβ
	35	Galβ1-4GlcNAcβ1-3Galβ1-4GlcNAcβ
	36	Galβ1-4GlcNAcβ1-6Galβ1-4GlcNAcβ
	37	Galβ1-4GlcNAcβ1-6(Galβ1-3)GalNAcα
	38	GalNacβ1-3GalαGalβ1-4Glcβ
	39	Galβ1-3GlcNAcβ1-3Galβ1-3GlcNAcβ
	40	Galα1-3Galβ1-4GlcNAcβ1-3Galβ1-4Glcβ
	41	Galβ1-4GlcNAcβ1-6(Galβ1-4GlcNAcβ1-3)GalNAcα
	42	Galβ1-4GlcNAcβ1-3(GlcNAcβ1-6)Galβ1-4GlcNAcβ
	43	Galβ1-4GlcNAcβ1-6(GlcNAcβ1-3)Galβ1-4GlcNAcβ
	44	(Galβ1-4GlcNAcβ1-3)3
	45	Galβ1-4GlcNAcβ1-6(Galβ1-4GlcNAcβ1-3)Galβ1-4GlcNAc
	46	Galβ1-3GalNAcβ1-3Galα1-4Galβ1-4Glcβ
	47	Galβ1-3GlcNAc
	48	Galβ1-4GlcNAc
	49	Galβ1-4Gal
	50	Galβ1-6GlcNAc
	51	Galβ1-3GlcNAc
	52	Galb1-3GalNAcβ1-4Galβ1-4Glc
	53	Galβ1-3GlcNAcβ1-3Galβ1-4Glc
	54	Galβ1-4GlcNAcβ1-3Galβ1-4Glc
	55	Galβ1-4GlcNAcβ1-6(Galβ1-4GlcNAcβ1-3)Galβ1-4Glc

	**56**	Galβ1-4GlcNAcβ1-6(Galβ1-3GlcNAcβ1-3)Galβ1-4Glc
	**57**	Galα1-4Galβ1-4Glc
	**58**	GalNAcα1-O-Ser
	**59**	Galβ1-3GalNAcα1-O-Ser
	**60**	Galα1-3Gal
	**61**	Galα1-3Galβ1-4GlcNAc
	**62**	Galα1-3Galβ1-4Glc
	**63**	Galα1-3Galβ1-4Galα1-3Gal
	**64**	Galβ1-6Gal
	**65**	GalNAcβ1-3Gal
	**66**	GalNAcβ1-4Gal
	**67**	Galα1-4Galβ1-4GlcNAc
	**68**	GalNAcα1-3Galβ1-4Glc
	**69**	Galβ1-3GlcNAcβ1-3Galβ1-4GlcNAcβ1-6(Galβ1-3GlcNAcβ1-3)Galβ1-4Glc
**Terminal glucose**	**70**	Glcα
	**71**	Glcβ
	**72**	GlcN(Gc)β
	**73**	HOCH2(HOCH)4CH2NH2
	**74**	GlcAα
	**75**	GlcAβ
	**76**	Glcα1-4Glcβ
	**77**	Glcβ1-4Glcβ
	**78**	Glcβ1-6Glcβ
	**79**	GlcAβ1-3GlcNAcβ
	**80**	GlcAβ1-3Galβ
	**81**	GlcAβ1-6Galβ
	**82**	(Glcα1-4)3β
	**83**	(Glcα1-6)3β
	**84**	(Glcα1-4)4β
	**85**	(Glcα1-6)4β
	**86**	(Glcα1-6)5β
	**87**	(Glcα1-6)6β
**Terminal N-acetyl glucosamine**	**88**	GalNAcα
	**89**	GlcNAcβ
	**90**	GlcNAcβ1-3GalNAcα
	**91**	GlcNAcβ1-4GlcNAcβ
	**92**	GlcNAcβ1-6GalNAcα
	**93**	GlcNAcβ1-4-[HOOC(CH3)CH]-3-O-GlcNAcβ
	**94**	GlcNAcβ1-[HOOC(CH3)CH]-3-OGlcNAcβ-L-alanyl-D-i-glutaminyl-L-lysine
	**95**	GlcNAcβ1-2Galβ1-3GalNAcα
	**96**	GlcNAcβ1-3Galβ1-3GalNAcα
	**97**	GlcNAcβ1-3Galβ1-4Glcβ
	**98**	GlcNAcβ1-3Galβ1-4GlcNAcβ
	**99**	GlcNAcβ1-4Galβ1-4GlcNAcβ
	**100**	GlcNAcβ1-6Galβ1-4GlcNAcβ
	**101**	GlcNAcβ1-6(Galβ1-3)GalNAcα
	**102**	GlcNAcβ1-6(GlcNAcβ1-3)GalNAcα
	**103**	GlcNAcβ1-6(GlcNAcβ1-3)Galβ1-4GlcNAcβ
	**104**	(GlcNAcβ1-4)5β
	**105**	(GlcNAcβ1-4)6β
	**106**	GlcNAcβ1-4GlcNAc
	**107**	GlcNAcβ1-4GlcNAcβ1-4GlcNAc
	**108**	GlcNAcβ1-4GlcNAcβ1-4GlcNAcβ1-4GlcNAc
	**109**	(GlcNAcβ1-4GlcNAc)3 β1-4
	**110**	GlcNAcβ1-4MurNAc
**Mannose-containing structures**	**111**	Manα
	**112**	Manβ
	**113**	ManNAcβ

	**114**	6-H2PO3Manα
	**115**	GlcNAcβ1-3Manβ
	**116**	Manβ1-4GlcNAcβ
	**117**	GlcNAcβ1-2Man
	**118**	GlcNAcβ1-2Manα1-6(GlcNAcβ1-2Manα1-3)Man
	**119**	Manα1-2Man
	**120**	120 Manα1-3Man
	**121**	121 Manα1-4Man
	**122**	122 Manα1-6Man
	**123**	123 Manα1-6(Manα1-3)Man
	**124**	Manα1-6(Manα1-3)Manα1-6(Manα1-3)Man
**Fucosylated structures**	**125**	Fucα
	**126**	Fucα1-3GlcNAcβ
	**127**	Fucα1-4GlcNAcβ
	**128**	Fucα1-2Galβ1-4GlcNAcβ
	**129**	Fucα1-2Galβ1-3GalNAcα
	**130**	Fucα1-2Galβ1-4Glcβ
	**131**	Galα1-3(Fucα1-2)Galβ
	**132**	Galα1-3(Fucα1-2)Galβ1-3GlcNAcβ
	**133**	Galα1-3(Fucα1-2)Galβ1-4GlcNAcβ
	**134**	Galα1-3(Fucα1-2)Galβ1-3GalNAcα
	**135**	Galα1-3(Fucα1-2)Galβ1-3GalNAcβ
	**136**	Galα1-3Galβ1-4(Fucα1-3)GlcNAcβ
	**137**	GalNAcα1-3(Fucα1-2)Galβ1-3GlcNAcβ
	**138**	GalNAcα1-3(Fucα1-2)Galβ1-4GlcNAcβ
	**139**	GalNAcα1-6(Fucα1-2)Galβ1-3GalNAcα
	**140**	Fucα1-2Galβ1-3GlcNAcβ1-3Galβ1-4GlcNAcβ
	**141**	Galα1-3(Galβ1-4)Fucα1-2(Fucα1-3)GlcNAcβ
	**142**	Fucα1-4(Fucα1-2Galβ1-3)GlcNAcβ1-3Galβ1-4Glcβ
	**143**	Fucα1-2Galβ1-4(Fucα1-3)GlcNAcβ1-3Galβ1-4Glcβ
	**144**	Lex1-6'(Lec1-3')Lac
	**145**	LacNAc1-6'(Led1-3')Lac
	**146**	Lex1-6'(6'SLN1-3')Lac
	**147**	Lex1-6'(Led1-3')Lac
	**148**	LecLex1-6'(Lec1-3')Lac
	**149**	Lex1-6'(Leb1-3')Lac
	**150**	Fucα1-2Galβ1-3GlcNAcβ1-3Galβ1-4Glc
	**151**	Galβ1-3(Fucα1-4)GlcNAcβ1-3Galβ1-4Glc
	**152**	Galβ1-4(Fucα1-3)GlcNAcβ1-3Galβ1-4Glc
	**153**	Fucα1-2Galβ1-3(Fucα1-4)GlcNAcβ1-3Galβ1-4Glc
	**154**	Galβ1-3(Fucα1-4)GlcNAcβ1-3Galβ1-4(Fucα1-3)Glc
	**155**	Fucα1-2Gal
	**156**	Fucα1-2Galβ1-4Glc
	**157**	Galβ1-4(Fucα1-3)Glc
	**158**	Galβ1-4(Fucα1-3)GlcNAc
	**159**	Galβ1-3(Fucα1-4)GlcNAc
	**160**	GalNAcα1-3(Fucα1-2)Gal
	**161**	Fucα1-2Galβ1-4(Fucα1-3)Glc
	**162**	Galβ1-3(Fucα1-2)Gal
	**163**	Fucα1-2Galβ1-4(Fucα1-3)GlcNAc
	**164**	Fucα1-2Galβ1-3GlcNAc
	**165**	Fucα1-2Galβ1-3(Fucα1-4)GlcNAc
	**166**	SO3-3Galβ1-3(Fucα1-4)GlcNAc
	**167**	SO3-3Galβ1-4(Fucα1-3)GlcNAc
	**168**	Galβ1-3GlcNAcβ1-3Galβ1-4(Fucα1-3)GlcNAcβ1-3Galβ1-4Glc
	**169**	Galβ1-4(Fucα1-3)GlcNAcβ1-6(Galβ1-3GlcNAcβ1-3)Galβ1-4Glc
	**170**	Galβ1-4(Fucα1-3)GlcNAcβ1-6(Fucα1-2Galβ1-3GlcNAcβ1-3)Galβ1-4Glc
	**171**	Galβ1-4(Fucα1-3)GlcNAcβ1-6(Fucα1-2Galβ1-3(Fucα1-4)GlcNAcβ1-3)Galβ1-4Glc

Siaylated structures	**172**	Neu5Acα
	**173**	Neu5Acα
	**174**	Neu5Gcα
	**175**	9-NAc-Neu5Acα
	**176**	Neu5Acα2-3Galβ
	**177**	Neu5Acα2-6Galβ
	**178**	Neu5Acα2-3GalNAcα
	**179**	Neu5Acα2-6GalNAcα
	**180**	Neu5Gcα2-6GalNAcα
	**181**	Neu5Acα2-8Neu5Acα2
	**182**	Neu5Acα2-6GalNAcβ
	**183**	Neu5Gcα2-3Gal
	**184**	Neu5Acα2-6(Galα1-3)GalNAcα
	**185**	Neu5Acα2-6(Galβ1-3)GalNAcα
	**186**	Neu5Acα2-3Galβ1-3GalNAcα
	**187**	Neu5Acα2-3Galβ1-3GlcNAcβ
	**188**	Neu5Gcα2-3Galβ1-4GlcNAcβ
	**189**	Neu5Gcα2-6Galβ1-4GlcNAcβ
	**190**	9-NAc-Neu5Acα2-6Galβ1-4GlcNAcβ
	**191**	Neu5Acα2-3Galβ1-4-(6-OSu)GlcNAcβ
	**192**	Neu5Acα2-3Galβ1-3-(6-OSu)GalNAcβ
	**193**	Neu5Acα2-6Galβ1-4-(6-OSu)GlcNAcβ
	**194**	Neu5Acα2-3-(6-O-Su)Galβ1-4GlcNAcβ
	**195**	(Neu5Acα2-8)3-sp3
	**196**	Neu5Acα2-6Galβ1-3GlcNAc-sp3
	**197**	Neu5Acα2-6Galβ1-3(6-OSu)GlcNAc
	**198**	Neu5Gcα2-3Galβ1-3GlcNAcβ-sp3
	**199**	GalNAcβ1-4(Neu5Acα2-3)Galβ1-4Glcβ
	**200**	Neu5Acα2-3Galβ1-4GlcNAcβ1-3Galβ
	**201**	Neu5Acα2-3Galβ1-4(Fucα1-3)6-OSu-GlcNAcβ
	**202**	Neu5Acα2-3(6-O-Su)Galβ1-4(Fucα1-3)GlcNAcβ
	**203**	Neu5Acα2-6(Neu5Acα2-3Galβ1-3)GalNAcα
	**204**	Neu5Acα2-8Neu5Acα2-3Galβ1-4Glcβ
	**205**	Neu5Acα2-3Galβ1-4GlcNAcβ1-3Galβ1-4GlcNAcβ
	**206**	Neu5Acα2-3 Galβ1-4(Fucα1-3)GlcNAcβ1-3Galβ
	**207**	Neu5Acα2-6(Galβ1-3)GlcNAcβ1-3Galβ1-4Glcβ
	**208**	GalNAcβ1-4(Neu5Acα2-8Neu5Acα2-3)Galβ1-4Glc
	**209**	Neu5Acα2-8Neu5Acα2-8Neu5Acα2-3Galβ1-4Glc
	**210**	GalNAcβ1-4(Neu5Acα2-8)2Neu5Acα2-3Galβ1-4Glc
	**211**	Neu5Acα2-3Galβ1-4GlcNAcβ1-3Galβ1-4GlcNAcβ
	**212**	Neu5Acα2-3Galβ1-4GlcNAcβ1-3Galβ1-4Glcβ-sp4
	**213**	Neu5Acα2-3Galβ1-3(Fucα1-4)GlcNAc
	**214**	Neu5Acα2-3Galβ1-4(Fucα1-3)GlcNAc
	**215**	Neu5Acα2-3Galβ1-3GlcNAcβ1-3Galβ1-4Glc
	**216**	Galβ1-4(Fucα1-3)GlcNAcβ1-6(Neu5Acα2-6Galβ1-4GlcNAcβ1-3)Galb1-4Glc
	**217**	Neu5Acα2-3Galβ1-3(Neu5Acα2-6)GalNAc
	**218**	Neu5Acα2-6Galβ1-3GlcNAcβ1-3Galβ1-4(Fucα1-3)Glc
	**219**	Neu5Acα2-3Galβ1-4GlcNAc
	**220**	Neu5Acα2-6Galβ1-4GlcNAc
	**221**	Neu5Acα2-3Galβ1-3GlcNAcβ1-3Galβ1-4Glc
	**222**	Galβ1-3(Neu5Acα2-6)GlcNAcβ1-3Galβ1-4Glc
	**223**	Neu5Acα2-6Galβ1-4GlcNAcβ1-3Galβ1-4Glc
	**224**	Neu5Acα2-3Galβ1-3(Neu5Acα2-6)GlcNAcβ1-3Galβ1-4Glc
	**225**	Neu5Acα2-3Galβ1-4Glc
	**226**	Neu5Acα2-6Galβ1-4Glc
	**227**	(Neu5Acα2-8Neu5Ac)n (n<50)
	**228**	Neu5Acα2-6Galβ1-4GlcNAcβ1-2Manα1-6(Neu5Acα2-6Galβ1-4GlcNAcβ1-2Manα1-6)Manβ1-
	**229**	3-O-Su-Galβ

Sulfated structures	**230**	230 3-O-Su-GalNAcα
	**231**	231 6-O-Su-GlcNAcβ
	**232**	232 3-O-Su-GlcNAcβ
	**233**	233 Galβ1-3(6-O-Su)GlcNAcβ
	**234**	234 Galβ1-4(6-O-Su)Glcβ
	**235**	235 Galβ1-4(6-O-Su)GlcNAcβ
	**236**	GlcNAcβ1-4(6-O-Su)GlcNAcβ
	**237**	3-O-Su-Galβ1-3GalNAcα
	**238**	6-O-Su-Galβ1-3GalNAcα
	**239**	3-O-Su-Galβ1-4Glcβ
	**240**	6-O-Su-Galβ1-4Glcβ
	**241**	3-O-Su-Galβ1-3GlcNAcβ
	**242**	3-O-Su-Galβ1-4GlcNAcβ
	**243**	4-O-Su-Galβ1-4GlcNAcβ
	**244**	6-O-Su-Galβ1-3GlcNAcβ
	**245**	6-O-Su-Galβ1-4GlcNAcβ
	**246**	3-O-Su-Galβ1-4(6-O-Su)Glcβ
	**247**	3-O-Su-Galβ1-4(6-O-Su)GlcNAcβ
	**248**	6-O-Su-Galβ1-4(6-O-Su)Glcβ
	**249**	6-O-Su-Galβ1-3(6-O-Su)GlcNAcβ
	**250**	6-O-Su-Galβ1-4(6-O-Su)GlcNAcβ
	**251**	3,4-O-Su2-Galβ1-4GlcNAcβ
	**252**	3,6-O-Su2-Galβ1-4GlcNAcβ
	**253**	4,6-O-Su2-Galβ1-4GlcNAcβ
	**254**	4,6-O-Su2-Galβ1-4GlcNAcβ
	**255**	3,6-O-Su2-Galβ1-4(6-OSu)GlcNAcβ
	**256**	GalNAcβ1-4(6-O-Su)GlcNAcβ
	**257**	3-O-Su-GalNAcβ1-4GlcNAcβ
	**258**	6-O-Su-GalNAcβ1-4GlcNAcβ
	**259**	6-O-Su-GalNAcβ1-4-(3-OSu)GlcNAcβ
	**260**	3-O-Su-GalNAcβ1-4(3-O-Su)-GlcNAcβ
	**261**	3,6-O-Su2-GalNAcβ1-4GlcNAcβ
	**262**	4,6-O-Su2-GalNAcβ1-4GlcNAcβ
	**263**	4,6-O-Su2-GalNAcβ1-4-(3-OAc)GlcNAcβ
	**264**	4-O-Su-GalNAcβ1-4GlcNAcβ
	**265**	3,4-O-Su2-Galβ1-4GlcNAcβ
	**266**	6-O-Su-GalNAcβ1-4(6-OSu)GlcNAcβ
	**267**	Galβ1-4(6-O-Su)GlcNAcβ
	**268**	4-O-Su-GalNAcβ1-4GlcNAcβ
	**269**	3-O-SuGalβ1-4GlcNAcβ1-3Galβ1-4GlcNAcβ
	**270**	4-O-SuGalβ1-4GlcNAcβ1-3Galβ1-4GlcNAcβ
Carrageen and GAG structures	**272**	(Sia2-6A-GN-M)2-3,6-M-GN-GNβ-sp4
	**273**	Neocarratetraose-41, 3-di-*O*sulphate (Na+)
	**274**	Neocarratetraose-41-*O*-sulphate (Na+)
	**275**	Neocarrahexaose-24,41, 3, 5-tetra-*O*-sulphate (Na+)
	**276**	Neocarrahexaose-41, 3, 5-tri-*O*sulphate (Na+)
	**277**	Neocarraoctaose-41, 3, 5, 7-tetra-*O*sulphate (Na+)
	**278**	Neocarradecaose-41, 3, 5, 7, 9-penta-*O*-sulphate (Na+)
	**279**	ΔUA-2S → GlcNS-6S Na4 (I-S)
	**280**	ΔUA → GlucNS-6S Na3 (II-S)
	**281**	ΔUA → 2S-GlcNS Na3 (III-S)
	**282**	ΔUA → 2S-GlcNAc-6S Na3 (I-A)
	**283**	ΔUA → GlcNAc-6S Na2 (II-A)
	**284**	ΔUA → 2S-GlcNAc Na2 (III-A)
	**285**	ΔUA → GlcNAc Na (IV-A)
	**298**	(GlcA/IdoAα/β1-4GlcNAcα1-4)n (n=200)
	**299**	(GlcA/IdoAβ1-3(±4/6S)GalNAcβ1-4)n (n<250)
	**300**	((±2S)GlcA/IdoAα/b1-3(±4S)GalNAcβ1-4)n (n<250)

	**301**	(GlcA/IdoAβ1-3(±6S)GalNAcβ1-4)n (n<250)
	**314**	(GlcA/IdoAα/ IdoASα/β1-4Glc*N*Ac/GlcNS/Glc*N*Ac6Sα1-4)n

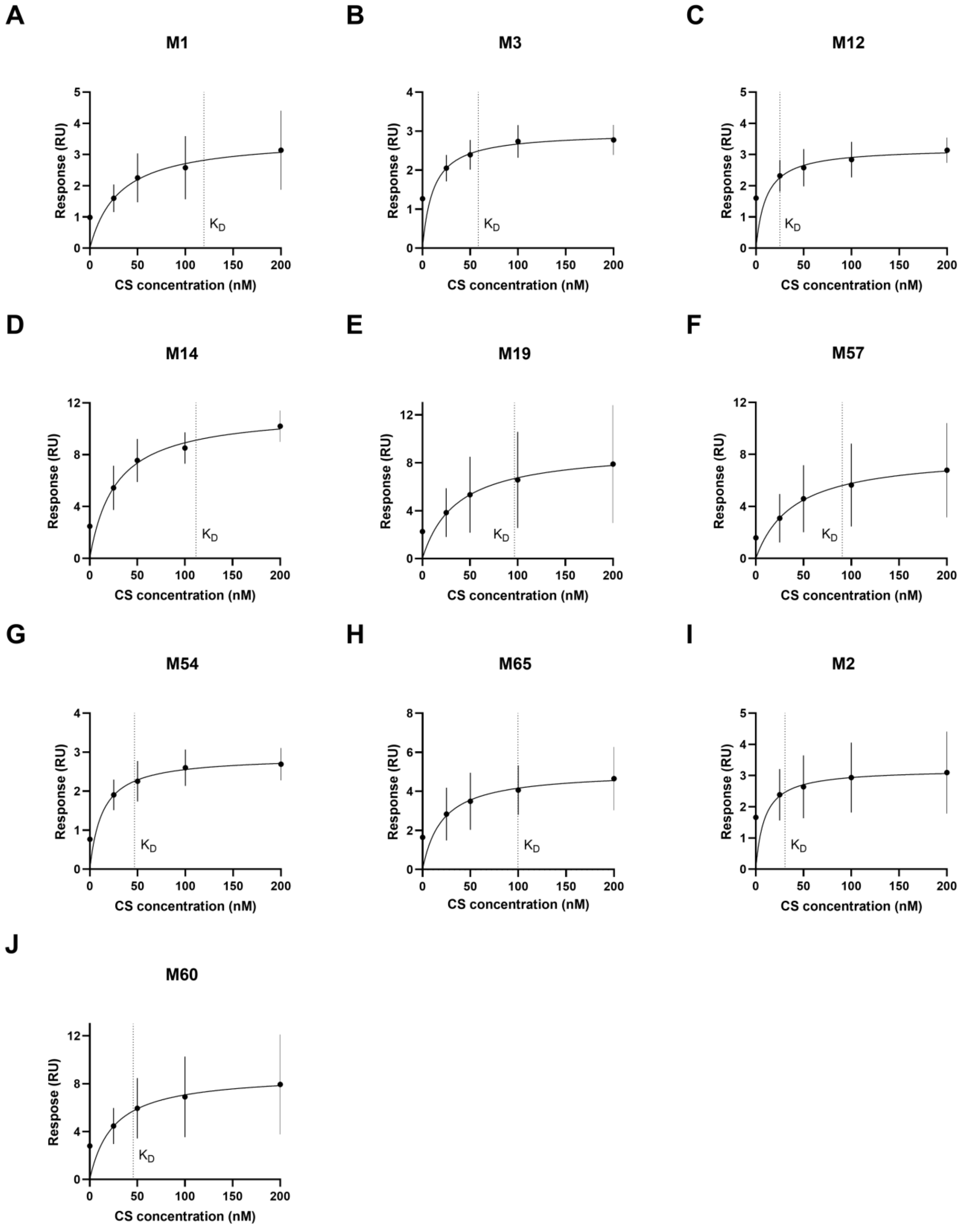
A
M1
Response (RU)
CS concentration (nM)
K_D
B
M3
Response (RU)
CS concentration (nM)
K_D
C
M12
Response (RU)
CS concentration (nM)
K_D
D
M14
Response (RU)
CS concentration (nM)
K_D
E
M19
Response (RU)
CS concentration (nM)
K_D
F
M57
Response (RU)
CS concentration (nM)
K_D
G
M54
Response (RU)
CS concentration (nM)
K_D
H
M65
Response (RU)
CS concentration (nM)
K_D
I
M2
Response (RU)
CS concentration (nM)
K_D
J
M60
Respose (RU)
CS concentration (nM)
K_D

Supplementary Fig. S1: Steady-state affinity curves of M protein – chondroitin sulfate interactions. (A-J) Affinity curves of chondroitin sulfate (CS) binding to (A-F) M proteins of A-C pattern strains, (G-H) M proteins of D pattern strains, and (I-J) M proteins of E-pattern strains. (A-J) Steady-state affinity analysis of M protein – chondroitin sulfate interactions using surface plasmon resonance (SPR) followed a 1:1 Langmuir binding model. Equilibrium dissociation constants (K_D) are marked. Data shown are mean ± SEM from three independent experiments.

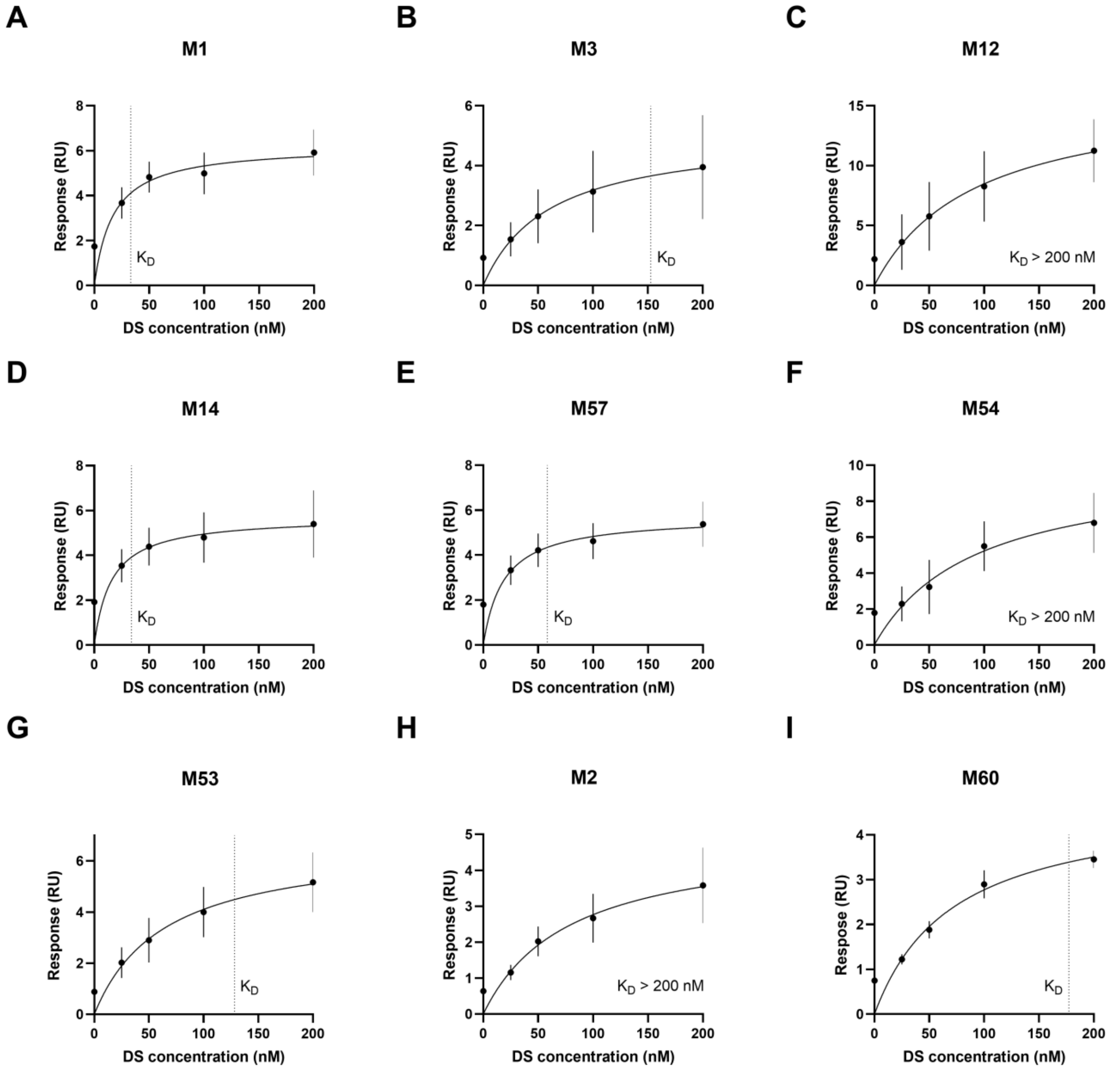

Supplementary Fig. S2: Steady-state affinity curves of M protein – dermatan sulfate interactions. (A-I) Affinity curves of dermatan sulfate (DS) binding to (A-E) M proteins of A-C pattern strains, (F-G) M proteins of D pattern strains, and (H-I) M proteins of E-pattern strains. (A-I) Steady-state affinity analysis of M protein – dermatan sulfate interactions using surface

plasmon resonance (SPR) followed a 1:1 Langmuir binding model. Equilibrium dissociation constants (K_D) are marked. Data shown are mean ± SEM from three independent experiments.

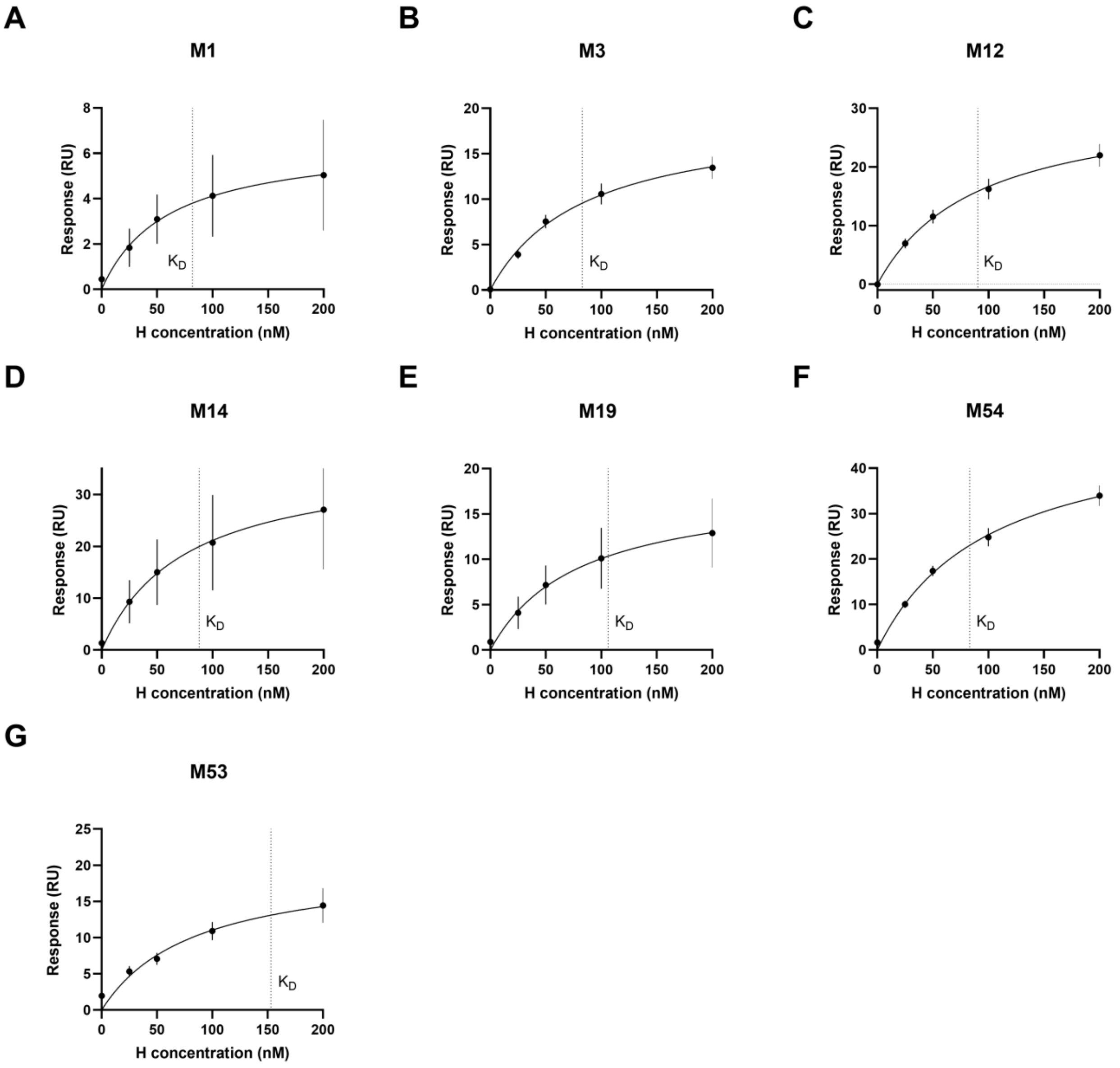

Supplementary Fig. S3: Steady-state affinity curves of M protein – heparin interactions. (A-G) Affinity curves of heparin (H) binding to (A-E) M proteins of A-C pattern strains and (F-G) M proteins of D pattern strains. (A-G) Steady-state affinity analysis of M protein – heparin interactions using surface plasmon resonance (SPR) followed a 1:1 Langmuir binding model.

Equilibrium dissociation constants (K_D) are marked. Data shown are mean ± SEM from three independent experiments.

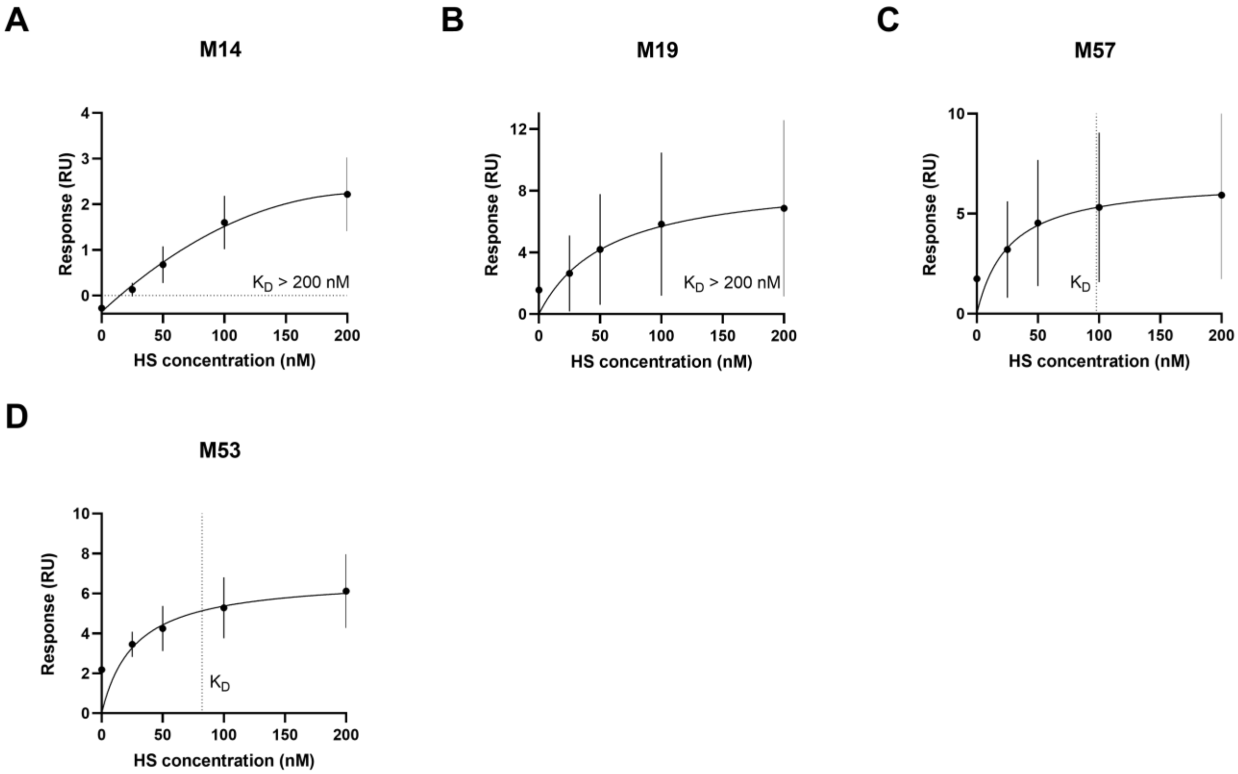

Supplementary Fig. S4: Steady-state affinity curves of M protein – heparan sulfate interactions. (A-D) Affinity curves of heparan sulfate (HS) binding to (A-C) M proteins of A-C pattern strains and (D) M53 of D pattern strains. (A-D) Steady-state affinity analysis of M protein – heparan sulfate interactions using surface plasmon resonance (SPR) followed a 1:1 Langmuir binding model. Equilibrium dissociation constants (K_D) are marked. Data shown are mean ± SEM from three independent experiments.

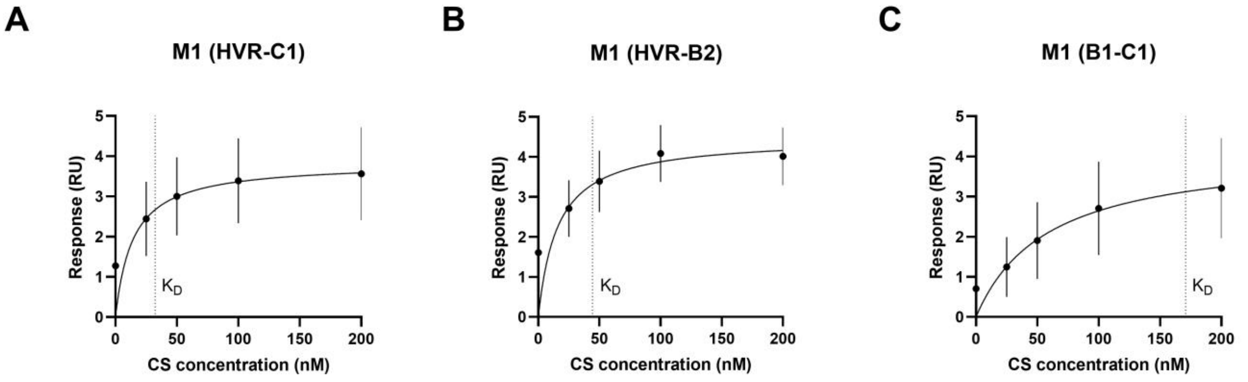

Supplementary Fig. S5: Steady-state affinity curves of M1 protein fragment – chondroitin sulfate interactions. (A-C) Affinity curves of chondroitin sulfate (CS) binding to M1 protein fragments. Steady-state affinity analysis of M1 protein fragment – chondroitin sulfate interactions using surface plasmon resonance (SPR) followed a 1:1 Langmuir binding model. Equilibrium dissociation constants (K_D) are marked. Data shown are mean ± SEM from three independent experiments.

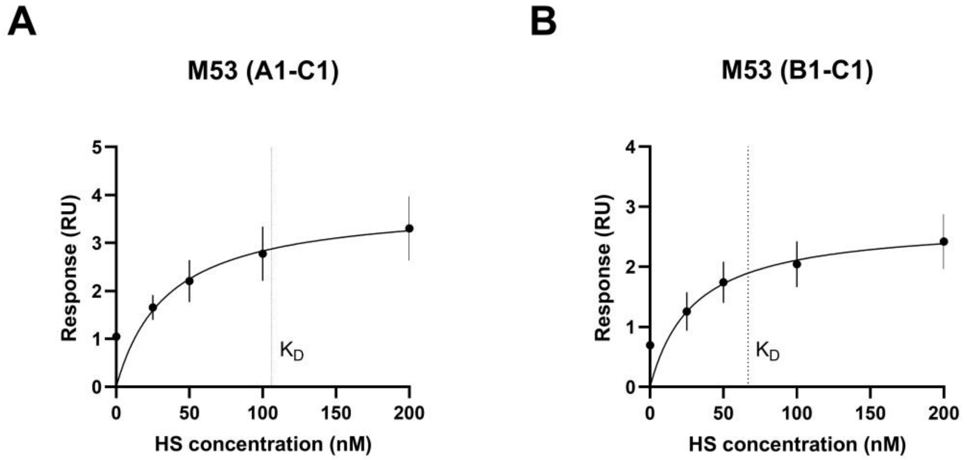

Supplementary Fig. S6: Steady-state affinity curves of M53 protein fragment – heparan sulfate interactions. (A-B) Affinity curves of heparan sulfate (HS) binding to M53 protein fragments. Steady-state affinity analysis of M53 protein fragment – heparan sulfate interactions using surface plasmon resonance (SPR) followed a 1:1 Langmuir binding model. Equilibrium dissociation constants (K_D) are marked. Data shown are mean ± SEM from three independent experiments.

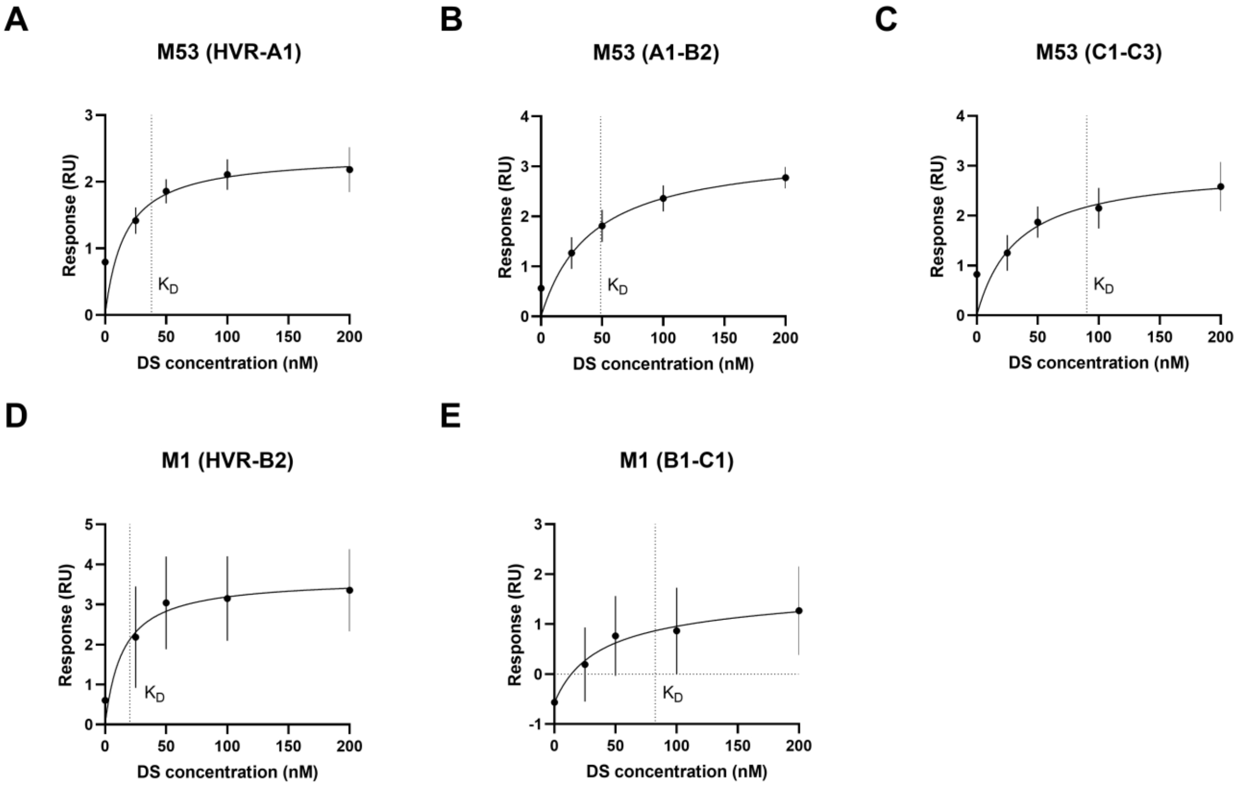

Supplementary Fig. S7: Steady-state affinity curves of M protein fragment – dermatan sulfate interactions. (A-E) Affinity curves of dermatan sulfate (DS) binding to (A-C) M53 protein fragments and (D-E) M1 protein fragments. (A-G) Steady-state affinity analysis of M protein fragment – dermatan sulfate interactions using surface plasmon resonance (SPR) followed a 1:1 Langmuir binding model. Equilibrium dissociation constants (K_D) are marked. Data shown are mean ± SEM from three independent experiments.

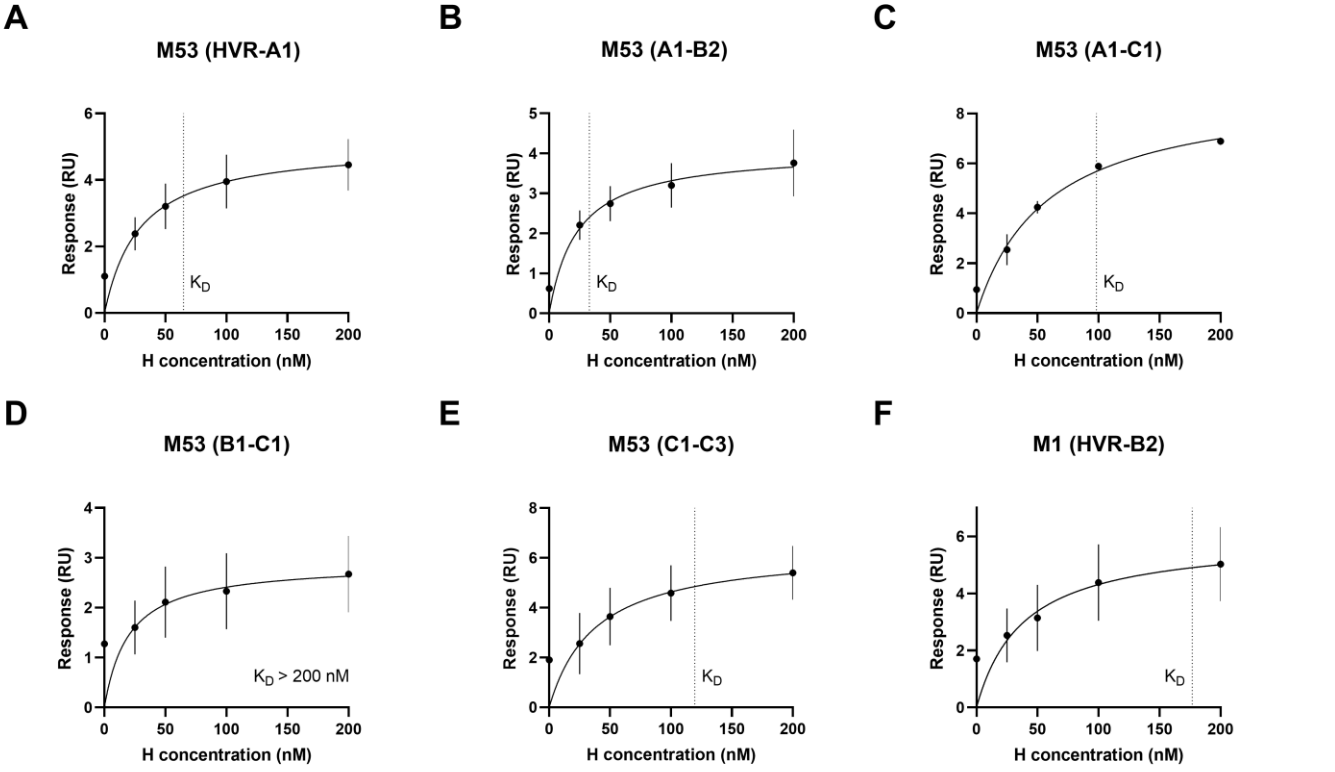

Supplementary Fig. S8: Steady-state affinity curves of M protein fragment – heparin interactions. (A-F) Affinity curves of heparin (H) binding to (A-E) M53 protein fragments and (F) M1 (HVR-B2) protein fragment. (A-F) Steady-state affinity analysis of M protein fragment – heparin interactions using surface plasmon resonance (SPR) followed a 1:1 Langmuir binding model. Equilibrium dissociation constants (K_D) are marked. Data shown are mean ± SEM from three independent experiments.

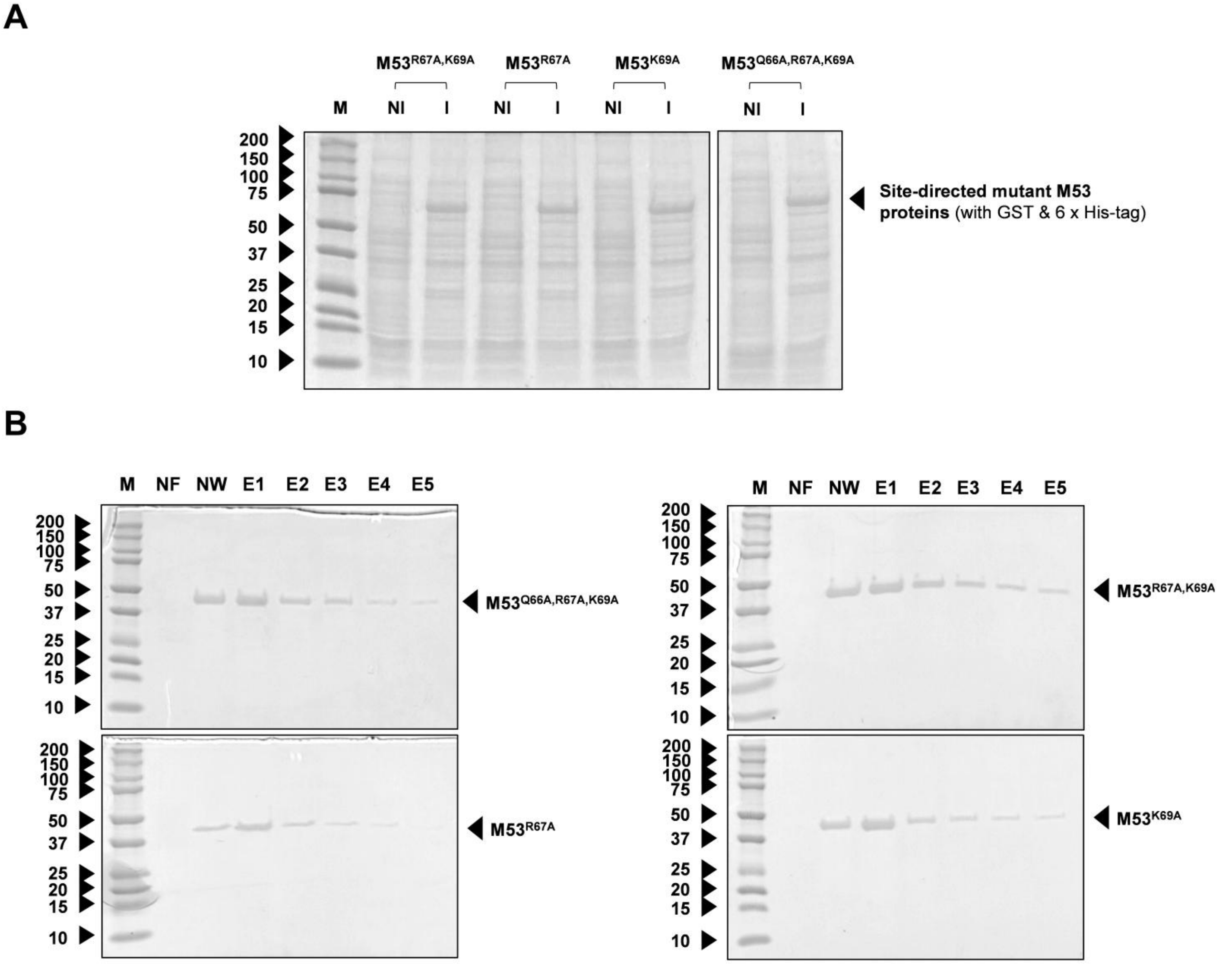

Supplementary Fig. S9: Expression and purification profile of site-directed mutant M53 proteins. (A) Protein expression was induced (I) in Top10 *E. coli* and assessed after 4-h compared with a non-induced (NI) sample. (B) M proteins were partially purified using glutathione-affinity chromatography via cleavage of the glutathione-S-transferase (GST) tag (not shown), followed by Ni^{2+} – NTA chromatography. Protein loss was assessed by sampling flow-through (NF) and column wash (NW). M protein elutions were collected in fractions (E1 – E5). (A-B) Samples (50

μL) were loaded 1:10 onto a 10% polyacrylamide gel and electrophoresed under reducing conditions and proteins were visualised using Rapid stain.
